# Evaluating the risk of dapsone hypersensitivity syndrome in Nepalese Leprosy Patients via HLA-B* 13:01 screening using real-time PCR and comparative meta-analysis of international data

**DOI:** 10.1371/journal.pntd.0014568

**Published:** 2026-08-13

**Authors:** Divya RSJB Rana, Mahesh Shah, Suwash Baral, Reejana Shrestha, Kishor Koju, Kanchha Shrestha, Preeti Maharjan, Jarina Joshi, Indra Bahadur Napit, Pushpendra Singh, Hana Krismawati, Deanna A. Hagge

**Affiliations:** 1 Mycobacterial Research Laboratory, Anandaban Hospital, The Leprosy Mission Nepal, Tikabhairab, Lele, Godawari, Lalitpur, Bagmati, Nepal; 2 Central Department of Biotechnology, Tribhuvan University, Kirtipur, Kathmandu, Bagmati, Nepal; 3 Anandaban Hospital, The Leprosy Mission Nepal, Tikabhairab, Lele, Godawari, Lalitpur, Bagmati, Nepal; 4 Medical Records, Anandaban Hospital, The Leprosy Mission Nepal, Tikabhairab, Lele, Godawari, Lalitpur, Bagmati, Nepal; 5 ICMR-National Institute of Research in Tribal Health, Jabalpur, Madhya Pradesh, India; 6 Academy of Scientific and Innovative Research (AcSIR), Ghaziabad, India; 7 Papua Agency, National Institute of Health Research and Development, Ministry of Health, Indonesia, Jayapura, Indonesia; University of Bremen: Universitat Bremen, GERMANY

## Abstract

**Background:**

Dapsone Hypersensitivity Syndrome (DHS) is a serious debilitating condition which can develop after 2–8 weeks of dapsone treatment in varying proportions between genetically diverse populations. Approximately 10% of the affected individuals die, and DHS patients often spend weeks to months in the hospital, which impacts health and psychological morbidity and household financial burden. In recent years, a human leukocyte antigen, HLA-B*13:01, has been consistently associated with up to 85% of DHS cases across international population studies; however, the necessity of next generation sequencing (NGS) severely limits clinical applications in low resource contexts.

**Methodology/Principal Finding:**

To investigate HLA-B*13:01 associations with DHS among Nepalese leprosy cases, retrospective and active DHS cases and dapsone-tolerant controls treated at least for 3 months with multi-drug therapy (MDT) were sampled and screened by HLA-B*13:01 qPCR. In the present study we enrolled 34 DHS cases and 82 dapsone tolerant controls and found that the association is maintained in a multi-ethnic Nepali population with an Odds Ratio of 50.1 (95% CI: 15.0-166.6). A previously validated qPCR-based commercial kit was used in the study, and we revalidated the methodology (23 negative and 35 positives by commercial qPCR) using Next Generation sequencing (NGS) method and found a concordance rate of 98.3%. We meta-analyzed all eligible HLA-B*13:01 and DHS association studies and found a summary Odds Ratio of 61.86 (95% CI 32.60 - 117.4). As 23.5% of the DHS cases were HLA-B*13:01 negative in our study, further analyses of the HLA-B*13:01 positive and negative study participants revealed that HLA-B*13:01 positive DHS cases were significantly younger than HLA-B*13:01 negative DHS cases (35.5 years vs. 66 years, p = 0.0018). The positive predictive value of the HLA test in the Nepalese population was ~ 24.

**Conclusion:**

The study validates the association between HLA-B*13:01 and DHS in Nepalese leprosy population. Inclusion of a genetic screening test before starting MDT could potentially prevent significant proportion of DHS occurring in leprosy cases, especially in South Asian and Southeast countries.

## Author summary

Dapsone, primarily used to treat leprosy, can trigger dapsone hypersensitivity (DHS) which is a severe debilitating, drug allergy episode lasting weeks to months in people after taking daily dapsone for 2–8 weeks. DHS can result in severe morbidity requiring prolonged hospitalization with mortality occurring in roughly 10%. The genetic locus HLA-B*13:01 is a significant risk factor and has been reported in various populations to be present in 80–90% of DHS cases. Prevalence of HLA-B*13:01 may vary in different ethnic or regional populations, and thus a qPCR-based study with DNA sequence validation was performed to estimate the association between DHS and HLA-B*13:01 Nepalese leprosy cases. In our analysis, we found that DHS cases about 50 times more likely to be HLA-B*13:01 positive than controls. The association in our study was found to be within the range of meta-analysis association based on population studies in India, China, Taiwan, Indonesia, Thailand, and South Korea. These findings indicate the efficacy of HLA-B*13:01 qPCR pre-screening before initiation of dapsone treatment to prevent most DHS development, and thereby, reduce associated catastrophic health and economic impacts.

## Introduction

Dapsone, discovered in the 1940s, was the first effective chemotherapeutic agent for leprosy [1]. In 1982, daily dapsone was combined with monthly rifampicin and clofazimine as part of multidrug therapy (MDT), which is still recommended by the World Health Organization for leprosy treatment today [2,3]. Among these drugs, monthly rifampicin is regarded as the safest with respect to adverse events, demonstrating minimal significant side effects. [4–6]. Clofazimine, commercially known as Lamprene, is also relatively safe but varies in tolerability due to skin darkening and dryness, which can fade away within months of MDT cessation [7].

However, dapsone is considered the least safe antibiotic in MDT due to potentially serious adverse events (SAE) of anemia, methemoglobinemia, and dapsone hypersensitivity syndrome (DHS) which has high morbidity and potential mortality [8–12]. In the literature, DHS is variably reported as dapsone syndrome, sulphone syndrome, dapsone allergy, or Drug Reaction with Eosinophilia and Systemic Syndrome (DRESS). DRESS is a nomenclature used by the European multinational registry for severe cutaneous adverse reactions (RegiSCAR) to describe drug-induced hypersensitivity that occurs within 2–8 weeks of drug treatment and is different from the other two severe cutaneous adverse reactions (SCARs) namely SJS/TEN (Steven Johnson Syndrome/ Toxic Epidermal Necrolysis) and AGEP Acute Generalized Exanthematous Pustulosis) [13].

While the patho-mechanisms of drug-induced hypersensitivities are unclear, there is considerable evidence of key factors potentially involved. Immunologically, drug-induced hypersensitivity reactions, such as DHS, are classified as Type IV hypersensitivity reactions according to the Gel and Coombs system [14–16]. Both CD4 and CD8 cells [15,17–19] as well as perforins, Granzyme B, and other pro-inflammatory cytokines [17,20–22] have been associated with dapsone-induced and other drug-induced hypersensitivity reactions. While an in vitro study found the ”p-i concept”, for pharmacological interaction of drugs with immune receptors, to be a more plausible mechanism for DHS [23], there are multiple possibilities that may be engaged. Dapsone can fit directly into the class I human leukocyte antigen HLA-B*13:01 peptide-binding cleft that leads to changes in immunopeptidome and stimulation of T cells [24, 25]. Other mechanisms like prohapten/hapten hypothesis may also be considered [26, 27].

DHS, like other drug-induced hypersensitivity reactions, is considered not to be dependent on dosage and usually presents with skin eruptions and systemic symptoms [16,28]. Occurring in an estimated 1–2% worldwide, DHS carries an estimated mortality risk of approximately 10%, which is comparable to other drug-induced hypersensitivity reactions, usually classified as DRESS [10,29]. Thus, DHS is more likely DRESS because of its involvement of systemic syndromes, including fever, lymphadenopathy, hepatitis, skin eruptions, and delayed time course initiating 2–8 weeks after drug intake, though SJS/TEN have also been reported [24, 30]. Presently, DHS is either defined as consisting of criteria as described by Richardus and Smith [10, 29] or by general DRESS criteria as defined by the RegiSCAR group (hereafter RegiSCAR criteria) in Europe [13]. The Japanese definition of DRESS adds one additional criterion of HHV-6 detection [31–33]. While HHV-6 association has not been systemically studied in DHS reactions, HHV-6 or HHV-5 reactivation is said to account for the increased severity of DRESS [31, 34, 35]. HHV-5 and/or HHV-6 were found in a significant proportion of DHS cases at our center [36].

In DHS-specific literature, DHS typically presents as a sudden severe illness within the first three months of dapsone/MDT treatment. DHS treatment involves discontinuing dapsone and managing symptoms with supportive care in an inpatient setting [37]. Patients generally remain hospitalized for weeks to months before being discharged to continue their recovery at home over the following months. DHS can have a severe and long-lasting impact on the patient’s life (health, school, employment, and relationships) as well as their household income and family dynamics. Therefore, preventive screening for DHS susceptibility is ideal.

Using DNA sequencing, the presence of the HLA-B*13:01 allele has been associated with high sensitivity and specificity for the detection of approximately 85% of DHS cases in various populations including China [38, 39], Taiwan [20], Thailand [30], Korea (HJ et al., 2020), India [40] and Indonesia [41]. While the prevalence of the HLA-B*13:01 allele in Nepal has not been previously reported, the frequency of HLA-B*13:01 in various ethnicities in India and China ranges from 0% to 24% according to the HLA Net Database [42], and Nepalese ethnicities also lie in the same geographical region.

However, DNA sequencing is not a practical method for the preventative screening of DHS susceptibility in most leprosy-relevant, resource-limited contexts due to its high cost, requisite expertise, and limited technology access. However, due in part to the COVID-19 pandemic, national clinical laboratory networks for real-time, quantitative PCR [qPCR) screening have been enhanced and are now more accessible and feasible, especially for tropical diseases in many low- to middle-income countries (LMIC) [63].

Nalagenetics, Singapore [41, 43] has developed a proprietary qPCR kit that could be used to provide HLA-B*13:01 screening results within a few hours to days (depending on contexts), enabling identification of individuals at higher risk for developing DHS in a timely manner for clinical application. Therefore, all new cases could potentially begin rifampicin and clofazimine treatment at the time of leprosy diagnosis, while dapsone administration could be postponed for a few days until qPCR results are available. Individuals who test positive for the HLA-B*13:01 allele can be advised not to take dapsone, which is indicated as an alternative treatment in WHO guidelines [2].

In a hospital-based study, the prevalence of DHS in Nepal was found to be around 2% with a mortality rate of ~ 10% [44]. Another study calculated the combined average prevalence of the hypersensitivity in Nepalese and Bangladeshi populations to be around 3% [Smith et al., 2004]. Over the past few years, there have been roughly 2–3,000 new leprosy cases diagnosed every year in Nepal [45]. Excluding those taking dapsone for non-leprosy conditions, preventative diagnosis of high risk for DHS by detection of the HLA-B*13:01 allele could hypothetically eliminate severe impacts in the lives and socio-economic costs for an estimated up to 90 DHS cases developed among annual leprosy cases and households in Nepal. Thus, a hospital-based case-control study was designed to retrospectively inspect the presence of HLA-B*13:01 allele in DHS cases and dapsone-tolerant cases.

In the present study, we aimed to correlate the occurrence of DHS to the presence of HLA-B*13:01 allele in Nepalese leprosy cases. A recently available proprietary qPCR assay for HLA-B*13:01 allele detection [41, 43] was used to screen confirmed DHS cases and control leprosy cases who tolerated dapsone for at least 3 months without development of DHS. Previous studies including a few meta-analyses [46–48] have estimated higher odds of DHS in HLA-B*13:01 positive cases; but the same allele might not be responsible in drug-induced hypersensitivities by the same drug in different ethnic populations [49]. Thus, given the similarity of Nepalese genetic background to that of neighboring Chinese and Indians [50], and reported association of the allele with DHS in both Chinese [38, 39] and Indian [40] populations, we hypothesized that the association also existed in Nepalese population. We aimed to estimate the odds of Nepalese DHS cases compared to dapsone-tolerant controls being HLA-B*13:01 positive. Detection of the HLA-B*13:01 allele by qPCR was then validated in a subset of samples by Next-Generation sequencing (NGS) at the HLA-B loci.

## Methods

### Ethics Statement

The study was approved by the Nepal Health Research Council (NHRC) with reference number 2391/2022, and all participants were enrolled via signed informed consent prior to sample collection.

**Fig 1:**
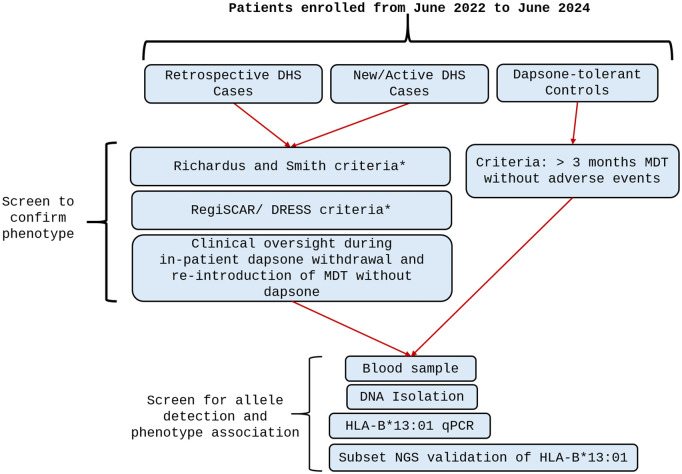
Study design for case and control enrollment.

Established in 1957 AD, the study site Anandaban Hospital in Lalitpur district Nepal, has been serving as non-profit, tertiary-level, national referral center for leprosy in Nepal; and the hospital also provides satellite clinics performed weekly or monthly in Kathmandu (Central Nepal), Butwal (Western Nepal), and Biratnagar (Eastern Nepal). Both historical or retrospective, and new or active DHS cases were enrolled in the study. The retrospective DHS patients, who were previously successfully treated for DHS, were recruited among those who presented at the hospital or clinics for leprosy-related complication management during the study period. Demographic and clinical data were collected from the patient charts and, when possible, from direct interviews with the retrospective and new DHS study participants. For new leprosy cases (described in another manuscript), results of preventative HLA-B*13:01 screening by qPCR were provided to the treating clinician and patients within 1 week of blood collection; and findings were added to their individual patient medical chart file. Thereby, results could guide for use of dapsone or any other sulfa drug, if required, in the future. The data from patient interviews and medical charts were recorded in hard copy Case Report Forms (CRFs) and later transcribed to Microsoft Excel files. Confidentiality was kept with study data accessible to the study team only. Study design is shown in Fig 1. The study is reported according to Strobe checklist (S1 Strobe checklist, www.strobe-statement.org)

### Leprosy diagnosis

All leprosy cases were diagnosed and treated as per standard World Health Organization (WHO) guidelines (WHO, 2018). For those diagnosed by Anandaban Hospital services, comprehensive leprosy diagnosis included slit skin smear/ bacterial index (BI) determination, skin biopsy histopathology, and assessment by physiotherapist for voluntary muscle testing and sensory testing (VMT/ST) and clinical examination [51]. All the consultant dermatologists involved in the present study had 8–30 years of experience in diagnosing and treating leprosy at Anandaban Hospital, including the management of DHS.

### Dapsone Hypersensitivity (DHS) Diagnosis

Diagnoses were confirmed by consultant dermatologists based on Richardus and Smith criteria [29] which defines DHS as having: 1) presence of at least two of the following signs or symptoms: fever, skin eruption, lymphadenopathy, and liver abnormalities (hepatomegaly, hepatitis, jaundice, and/or abnormal liver function tests); 2) appearance of adverse events in the interval between the second and eighth week from start of dapsone administration, which subsided upon discontinuation of the drug; 3) symptoms not attributable to any other simultaneously used drugs or to leprosy reactions; and 4) symptoms not attributable to any other diseases. Additionally, RegiSCAR criteria were used to score the DHS episode whenever all lab and clinical data were available for the individual study participants [13]. RegiSCAR criteria is based on a set of clinical and laboratory items: fever, eosinophilia, atypical lymphocyte, skin involvement, other organ involvement, resolution time, and evaluation of other suspicious causes among others. The probability of the episode being considered as DRESS depends on the score obtained: final score < 2 as not DRESS, 2–3 as possible, 4–5 as probable, and > 5 as definite. Both Richardus and Smith criteria and RegiSCAR criteria were applied to all DHS cases as per data available.

All DHS cases at the study site, irrespective of their inclusion in the present study, undergo rifampicin re-challenge after recovery under active clinical and laboratory surveillance as part of routine institutional practice. After confirmation of absence of hypersensitivity to rifampicin, all DHS cases are treated with monthly rifampicin and daily clofazimine and/or other alternative regimens according to WHO recommendations [2]. This further confirmed that the hypersensitivity observed in our cases were attributed to dapsone rather than rifampicin and clofazimine.

### DHS cases and Dapsone-Tolerant controls

New (or active) and retrospective (those with previously confirmed) DHS patients were enrolled as cases. Newly diagnosed cases were those recently diagnosed with DHS, but the hypersensitivity symptoms were still unresolved at the time of sample collection. Newly diagnosed cases could be those who were registered and had initiated their MDT from the study site (Anandaban Hospital or satellite clinics) or those who were diagnosed and their MDT commenced elsewhere but referred to Anandaban Hospital for further management. All DHS cases were treated with steroids which usually consisted of dexamethasone injection followed by tapering dosages of prednisolone [37, 52]. Retrospective DHS cases included leprosy patients registered at Anandaban Hospital or elsewhere, but the DHS symptoms and sequelae had already resolved at the time of sample collection. Medical charts of each of the retrospective DHS cases were reviewed by senior study clinicians to align with Richardus and Smith criteria which also led to exclusion of cases where definitive DHS status could be conflicted with symptoms of other dapsone intolerances (e.g., methemoglobinemia) or leprosy reactions. Dapsone-tolerant control cases included those registered at Anandaban Hospital who had completed at least 3 months of daily dapsone as per standard MDT for leprosy. Leprosy cases were not enrolled if their dapsone was discontinued due to other adverse events such as anemia, methemoglobinemia, or known G6PD deficiency.

### Sample collection and DNA extraction

Up to three ml blood samples were collected in EDTA vials and stored at -20ºC until processed. Genomic DNA (gDNA) was extracted with Qiagen’s DNeasy Blood and Tissue Kit (Cat No./ID: 69506) according to manufacturer’s protocol. In brief, 100 µl of blood was mixed with 20 µl Proteinase K, 100 µl of PBS and 200 µl of AL buffer in 2.0 ml Eppendorf tubes, vortexed and incubated at 56ºC for 10 mins. After incubation, 200 µl of molecular grade absolute ethanol was added to the mixture, vortexed and transferred to DNeasy Mini spin column to spin at 6000xg for 1 min. Then the column was washed once with AW1 at 6000xg for 1 min followed by another wash with AW2 at 20,000xg/3 mins; and finally, the DNA was eluted with 200 µl AE buffer by spinning at 6000xg for 1 min. The extracted DNA was then stored at 4ºC until qPCR analyses.

### Quantitative PCR (qPCR)

DNA samples were tested for the presence of HLA-B*13:01 using a commercial kit Nala PGx 1301 Kit (Catalog N1301-100) from Nalagenetics, Singapore with the included mastermix. Due to pandemic supply chain shortages, some qPCR were later performed with a similar mastermix from the Qiagen Quantitect Probe PCR kit (Catalog 204343, Germany) after in-house validation against Nalagenetics mastermix. Reactions were run in 10 µl reaction volumes using 5 µl 2x mastermix, 1.6 µl primer probe mix and 2 µl gDNA and 1.4 µl nuclease-free water. The reactions were run for 15 minutes at 95°C followed by 40 cycles of 94°C for 10 secs and 61.5°C for 30 secs as mentioned in the manufacturer’s protocol using a Qiagen Rotor-Gene Q 5Plex HRM qPCR machine. The curve for beta actin was observed through red window (HEX dye) and HLA-B*13:01 was observed through green (FAM dye). All HLA-B*13:01 positive samples were rerun once again to confirm positivity. Any run with an aberrant PCR curve or negative beta actin curve was considered invalid, and the PCR reaction was performed again. All qPCR runs consisted of one extraction control from DNA extraction stage, one no template control, and one positive control provided in the kit. The cycle threshold (Ct) values for both HLA-B*13:01 and beta-actin were within 20–25 for both Nalagenetics or the alternative Qiagen Quantitect Probe PCR qPCR mastermix.

### Validation of qPCR Data

As a representative subset, gDNA samples whose qPCR tests were performed during the study period at the study site were sent for NGS-based typing. Fifty-eight (n = 58) gDNA samples found to be either HLA-B*13:01 positive or negative by the Nalagenetics qPCR method were sent to GenDx in the Netherlands for four-field resolution of HLA-B locus alleles using NGS. These included 28 samples from participants enrolled in the present study (23 cases and 5 controls) and 30 samples as an independent validation cohort. NGSgo-AmpX v2 HLA-B kit was used for the target generation and the NGSgo-Library Full Kit was used for the library preparation. Sequencing was done using the illumina MiSeq system. Analysis was performed in NGSengine (version 3.1.2.34113, IMGT/HLA 3.56.0). NGSgo Workflow for Illumina was used for generation of HLA-typing results. NGSengine was used for analysis of NGS data. HLA Sequences were identified using the International ImMunoGeneTics/Human Leukocyte Antigen (IMGT/HLA) 3.56.0 database (https://www.ebi.ac.uk/ipd/imgt/hla/).

### Statistical calculations

In the literature, the reported proportion of HLA-B*13:01 positivity in DHS cases was 80–90%, and the proportion among DHS negative cases was 0–15% [46, 47]. Considering a conservative estimate of prevalence of only 80% allele positivity in cases and up to 20% in DHS controls, a 20/20 case/control sample size provided power of 99% at 5% level of significance (https://www.stat.ubc.ca/~rollin/stats/ssize/b2.html). Association between HLA-B*13:01 and DHS was estimated in odds ratio (OR). The OR with corresponding 95% confidence intervals (95% CI) was calculated by using Fisher’s exact test in GraphPad Prism 5. Matching of case and control age, sex, bacillary index (BI), sampling period, ethnicity were tested with Fisher’s exact test and Mann Whitney U at 0.05 level significance. Comparison of variables for HLA-B13:01 positive and negative DHS cases were performed similarly.

### Meta-analysis of DHS and HLA-B*13:01 association studies

A consolidated meta-analysis was carried out to estimate the association between HLA-B*13:01 and DHS. Studies associating the human leukocyte antigen allele HLA-B*13:01 with DHS were searched in PubMed and Embase databases. Additionally, Google searches were also performed to identify any related studies. PubMed was searched using the term “(((dapsone hypersensitivity) OR (sulfone allergy)) OR (dapsone allergy)) AND ((HLA-B*13:01) OR (HLA-B* 13:01))”. Embase was searched with the term: (dapsone AND hypersensitivity) OR (sulfone AND allergy) OR (dapsone AND allergy)) AND ((’hla-b13:01’). Meta-analysis was performed using the “meta” package in R statistical software with RStudio 2023.12.0 Build 369. The inverse-variance method was used for Fixed/Common effect, and Restricted Maximum Likelihood was used for Random effects models. The heterogeneity was assessed based on I-squared and Tau-squared tests. Details of the meta-analysis according to PRISMA guidelines [53] are provided in the supporting information (S2 File, S1 Fig). The meta-analysis included studies which enrolled dapsone-tolerant control populations rather than population controls. Risk of bias was estimated with the Newcastle-Ottawa scale (NOS) [54, 55]. The total NOS (max 9, min 0) risk score was used to categorize studies as Good, Fair and Poor quality based on the Agency for Healthcare Research and Quality (AHRQ) method [56]. A Begg’s Rank correlation test was used to estimate the publication bias [57].

## Results

### HLA-B*13:01 is associated with DHS in Nepal

**Fig 2:**
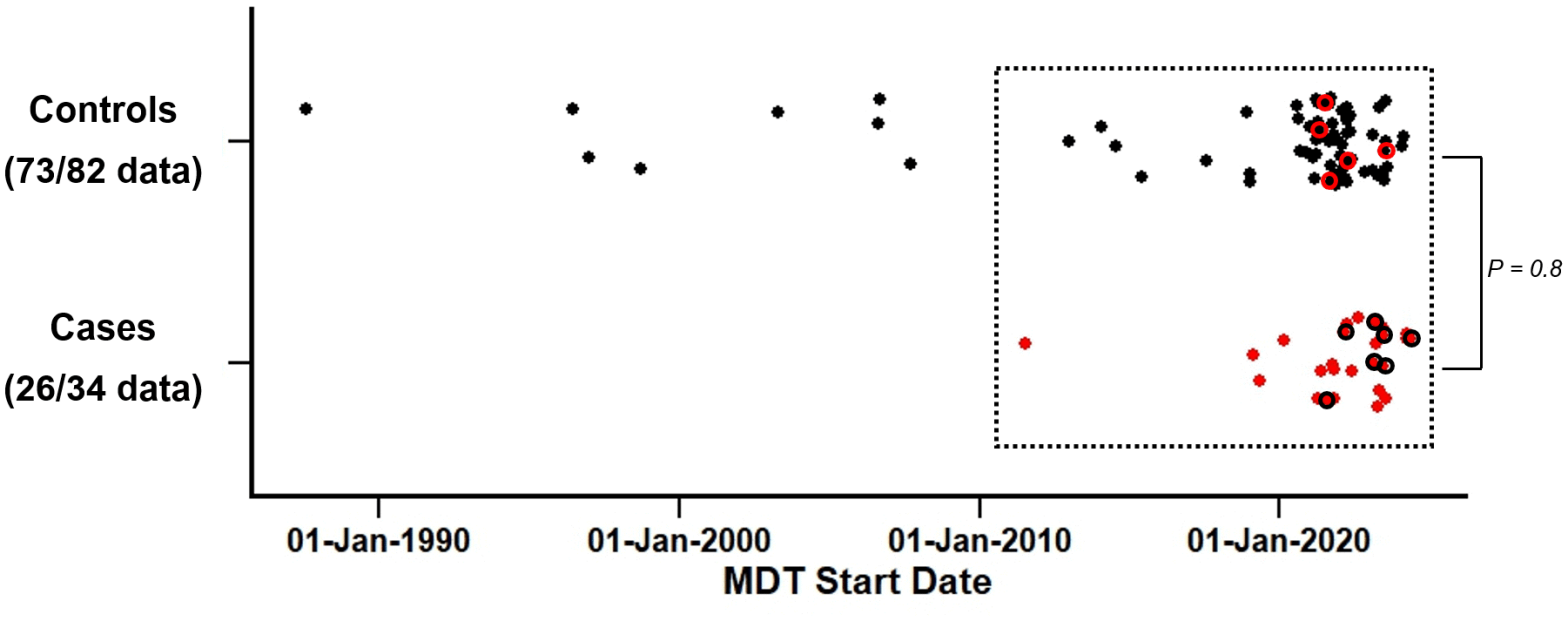
Distribution of MDT start dates in Case and Control groups. Black and Red dots denote control and case study participants respectively. Black and red circles denote HLA-B*13:01 negative DHS cases and HLA-B*13:01 positive dapsone-tolerant controls respectively. Analysis was based on data available, 26/34 for cases and 73/82 for controls.

**Fig 3:**
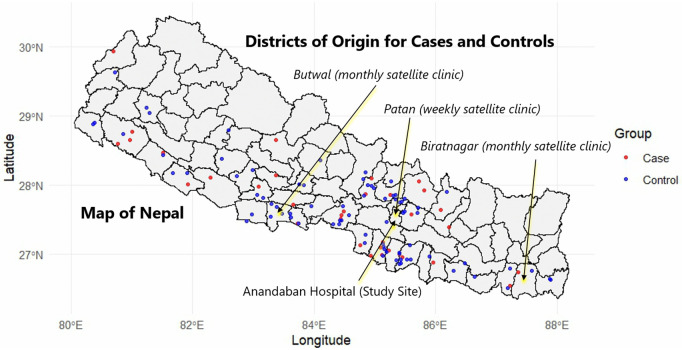
Districts of origin for DHS cases and dapsone-tolerant controls. Red dots depict DHS cases, blue dots depict controls. Map was drawn with RStudio 2023.12.0 Build 369, 2009-2023 Posit Software, PBC using “sf” (Simple Features) package. District level shape file of Nepal was downloaded from Humanitarian Data Exchange (https://data.humdata.org/dataset/cod-ab-npl) under the Creative Commons Attribution 4.0 International (CC BY 4.0) license.

In total, 34 newly or previously (retrospective) diagnosed DHS cases and 82 dapsone-tolerant controls were enrolled in the study between June 2022 and June 2024. Fifty-six percent (19/34) of the cases and 60% (49/82) of the controls were males (p = 0.84). The median age for cases was 39 years (range 15–78 years), which was similar to the median of 40 years (range 16–78 years) for controls (p = 0.54). Demographic characteristics of cases and controls are provided in Table 1. For DHS cases, MDT start date was available for 77% (26/34) of the cases, and ranged from July 2011 to May 2024. For controls, an exact MDT start date was available for 89% (73/82) of the participants, and ranged from July 1987 to February 2024. The average time since the control participants started their MDT at the time of enrollment was 54 months (range 3–480 months), with at least 60% having completed MDT at the time of sampling. All control participants received fixed-duration MDT, with dapsone, according to the WHO guidelines [2,58]. Eighty-nine percent (65/73) of these controls were from the same period as the DHS cases (p = 0.82) (Fig 2). Slit Skin Smear BI was available for 97.1% (33/34) of DHS cases and 91.5% (75/82) for controls; and 50% of DHS cases (17/34) and 58.5% (48/82) of controls were BI positive (p = 0.28). Both the DHS and control participants enrolled in the study originated from far Eastern to far Western regions of Nepal with the highest representation from central Bagmati province and lowest from the Karnali province (Fig 3). Regarding ethnicity within Nepal, Brahmin/Chhetri, Janajati and Madhesis accounted for the majority of the cases and controls (matching, p = 0.06). Both cases (p = 0.43) and controls (0.53) had similar proportions of ethnicities compared to Nepali 2021 Census populations [59].

**Table 1:** Demographic Characteristics of DHS Cases and Dapsone-tolerant Controls.

Details	DHS Cases	Dapsone-tolerant	p-values
Sample Size	34	82	
Male (%)	19 (56%)	49 (60%)	0.84
Female (%)	15 (44%)	33 (40%)	
Median Age (Range)	39 years (15-78 years)	40 years (16-78 years)	0.54
BI: Negative (%), positive (%), N/A (%)	16/34 (47%), 17/34 (50%), 1/34 (3%)	27/82 (33%), 48/82 (59%), 7/82 (9%)	0.28
Ethnicities			
Brahmin/Chhetri	11 (32%)	28 (34%)	0.06
Janajati	12 (35%)	16 (20%)	
Madhesi	7 (21%)	11 (13%)	
Tharu	3 (9%)	8 (10%)	
Newar	1 (3%)	6 (7%)	
Dalit	0 (0%)	10 (12%)	
Muslim (considered ethnicity in Nepal)	0 (0%)	2 (2%)	
Others	0 (0%)	1 (1%)	

**Fig 4:**
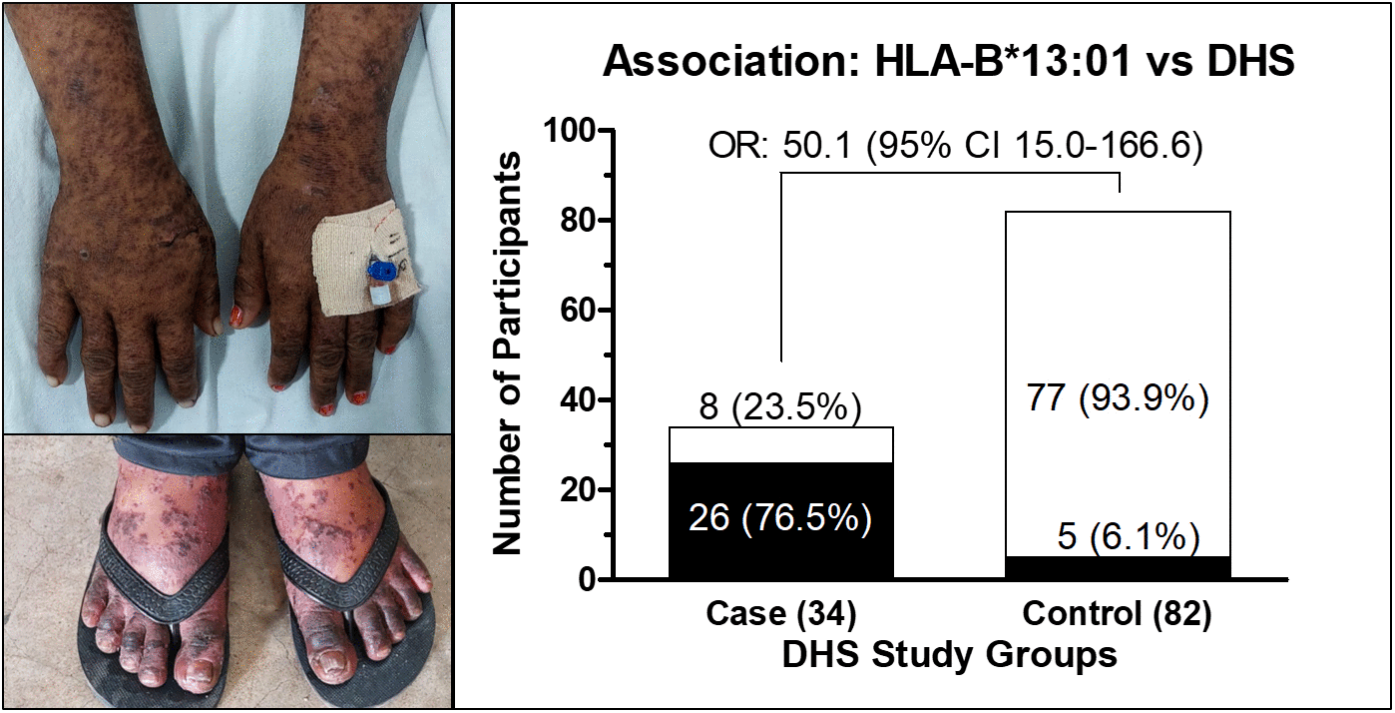
Prevalence of HLA-B*13:01 in DHS cases and Dapsone-tolerant Controls. The pictures are from 2 DHS participants, both in their twenties, a woman’s hands (above) and man’s feet (below).

Both Richardus and Smith criteria and RegiSCAR criteria were used to screen new and retrospective/historical DHS cases. Nine and ten DHS cases did not have enough data for the application of the Richardus and Smith, and the RegiSCAR criteria respectively. With regard to Richardus and Smith criteria, only 84% (21/25) of cases had symptoms within ≤ 8 weeks of dapsone start. Ninety-two percent (22/24) had fever, 95% (21/22) had skin symptoms, and 100% (25/25) had liver abnormalities. With regard to RegiSCAR criteria, 4% (1/24) of the case were categorized as possible, 83% (20/24) cases as probable and 13% (3/24) as definite DRESS. 76.5% (26/34) of DHS cases were positive for HLA-B*13:01, whereas only 6.1% (5/82) of controls had the allele (Fig 4, Table 2). The sensitivity of the test in confirmed DHS cases was 76.5% (95% CI: 58.8-89.3%) and the specificity for the controls was 93.9% (95% CI: 86.3-98.0%). Considering a population DHS incidence rate of 2.5%, the positive predictive value was 24.3% (95% CI: 11.9-43.4%) and negative predictive value was 99.4% (95% CI: 98.8-99.7%). The calculated OR for this association was 50.1 (95% CI: 15.0-166.6, p < 0.0001). Details of demographic, clinical and HLA typing data are provided in S1 File.

**Table 2:** Genotype Results by qPCR for DHS Cases and dapsone-tolerant Controls.

HLA-B*13:01 Typing		DHS Cases	Dapsone-tolerant Controls
HLA-B*13:01 by qPCR	*Positive*	26 *(76.5%)*	5 *(6.1%)*
	*Negative*	8 *(23.5%)*	77 *(93.9%)*
Total		34 *(100%)*	82 *(100%)*

### Meta-analysis

**Fig 5:**
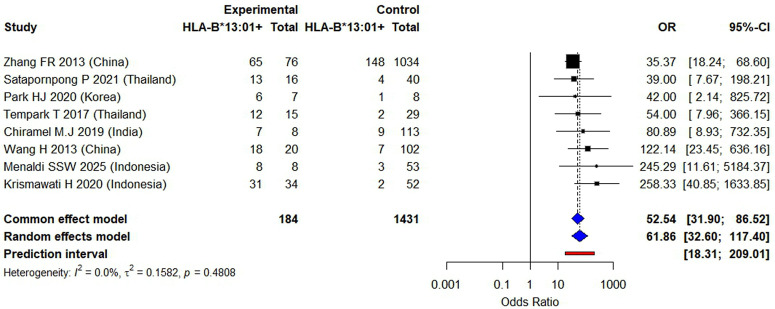
Meta-analysis for published DHS and HLA-B*13:01 association studies.

Since 2013 when the first two studies associating DHS with HLA-B*13:01 were published, many other research groups, particularly from South-East Asia, have validated the findings in their populations. To summarize the effect size of this association, a consolidated meta-analysis was performed (S2 File). All available research studies that included dapsone-tolerant patients as controls to compare against the DHS cases were included in a meta-analysis irrespective of DHS variants (DRESS and/or SJS/TEN) (S1 Table, S1 Fig). Two hundred and eighty five studies were retrieved for the analyses (104 from PubMed, 180 from Embase and 1 extra from Google search). Upon screening using the inclusion/exclusion criteria, eight studies were finally selected for meta-analysis, two each from China [38, 39], Indonesia [41], Menaldi 2025 [60] and Thailand [24, 30] and one each from South Korea [46] and India [40]. According to the Newcastle-Ottawa Scale Risk of Bias assessment (S2 Table), 7 studies were considered of Good quality and one of Fair quality [56]. Heterogeneity (tau^2 = 0.1582 (95% CI: 0.0000 - 1.6349]; I^2 = 0.0% (95% CI: 0.0%; 67.6%); Cochran’s Q = 6.52, df = 7, p = 0.4808) measures were not significant. The estimated summary ORs by Random and Fixed (Common) Effect Models were 61.86 (95% CI 32.60 - 117.4, p < 0.0001) and 52.54 (95% CI 31.90 - 86.52, p < 0.0001) respectively (S2 Fig). ORs for individual studies are provided in S3 Table. We consider the Random Effects Model more accurate because the study included in meta-analysis came from different geographical backgrounds. No publication bias (Begg’s test p-value 0.32) was detected (S3 Fig). The OR for our study, 50.1, was within the confidence interval of our own meta-analysis and previously reported meta-analytic studies [46, 47] (Fig 5).

### Validation of Nalagenetics HLA-B*13:01 qPCR detection in Nepalese by NGS

To validate the results of the commercial qPCR assay used in our study, a set of 58 genomic DNA samples, whose HLA-B*13:01 status were characterized at the study site, were sent for NGS-based 4-field typing of the HLA-B locus at GenDx in the Netherlands (Table 3). Twenty-three of them were the cases enrolled in the present study (20 HLA-B*13:01 positive and 3 negative), 5 were dapsone-tolerant controls (all HLA-B*13:01 positive), and 30 were an independent validation cohort (10 HLA-B*13:01 positive and 20 negative). There was 98.3% concordance between NGS and qPCR with all the 23 HLA-B*13:01 qPCR-negative samples confirmed as negative by NGS, and only one out of the 35 qPCR-positive samples indicative of a mismatch. The one mismatch was due to the allele HLA-B*13:148 identified as the best matching genotype by NGS, originating from a DHS case reported as HLA-B*13:01 positive by our qPCR test. The HLA-B*13:148 fell under same HLA-B*13:01G and HLA-B*13:01P groups which meant the genomic/DNA and peptide sequence in the peptide binding groove were identical for HLA-B*13:148 (IPD/IMGT HLA Database accession: MT902143) and HLA-B*13:01 (accession: D50290). There was only one nucleotide difference in the 52nd nucleotide of the coding DNA sequence (G52T) which caused one amino acid change (A18S). This amino acid change was present in exon 1 which does not participate in the peptide binding groove (formed by exons 2 and 3 only).

**Table 3:** Concordance between qPCR vs NGS testing for HLA-B*13:01.

		NGS-B locus	
		HLAB13:01 (+)	HLAB1301 (-)
**Nalagenetics qPCR kit**	HLA-B*13:01 (+)	34	1*
	HLA-B*13:01 (-)	0	23

*One sample was qPCR positive for HLA-B*13:01 but was HLA-B13:148 positive by NGS

Only one of the 23 DHS cases tested by NGS had homozygotic HLA-B*13:01 positivity (Table 4). She was a 43 year old female with a RegiSCAR DRESS score of 5 categorized as probable. There were a total of 15 probable cases analyzed by NGS among which one was homozygotic HLA-B*13:01 positive, 12 were heterozygotic HLA-B*13:01 positive and two were HLA-B*13:01 negative (one HLA-B*15:05:01:01/ 48:01:01:01 and one HLA-B*07:02:01:01/ 52:01:01:01). One definite (RegiSCAR) DHS case was HLA-B*13:01 negative (HLA-B* 40:01:02:01/ 51:06:01:01). All five control cases tested HLA-B*13:01 positive by qPCR were also confirmed by NGS (all heterozygotic).

**Table 4:** Genotyping results by Next Generation Sequencing for HLA-B*13:01 positive and negative results of qPCR. See text for details.

SN	HLA-B*13:01 qPCR	DRESS Category	Patient Type	Age	Sex	Result1	HLA-B Allele 1	HLA-B Allele 2
1	Negative	Probable	Case	68	F	15:05:01:01	48:01:01:01	Yes
2	Negative	na*	Independent^#^	40	M	08:01:01:02	58:01:01:01	Yes
3	Negative	na	Independent	29	M	35:03:01:01	40:26	Yes
4	Negative	na	Independent	65	F	57:01:01:01	58:01:01:01	Yes
5	Negative	na	Independent	17	F	35:01:01:02	38:01:01:01	Yes
6	Negative	na	Independent	24	F	38:02:01:01	51:08:01:01	Yes
7	Negative	na	Independent	41	F	18:01:01:02	35:01:01:02	Yes
8	Negative	Probable	Case	55	M	07:02:01:01	52:01:01:01	Yes
9	Negative	Definite	Case	72	F	40:01:02:01	51:06:01:01	Yes
10	Negative	na	Independent	26	M	15:25:01:01	38:02:01:01	Yes
11	Negative	na	Independent	69	F	35:01:01:02	38:02:01:01	Yes
12	Negative	na	Independent	44	F	15:18:01:02	57:01:01:01	Yes
13	***Positive***	Probable	Case	45	M	***13:01:01:01***	35:01:01:02	Yes
14	***Positive***	Probable	Case	26	M	***13:01:01:01***	48:01:01:01	Yes
15	***Positive***	na	Independent	24	M	***13:01:01:01***	55:01:01:01	Yes
16	***Positive***	Probable	Case	68	M	***13:01:01:01***	51:06:01:01	Yes
17	***Positive***	No Data^&^	Case	22	M	***13:01:01:01***	40:06:01:02	Yes
18	***Positive***	Probable	Case	43	F	***13:01:01:01***	***13:01:01:01***	Yes
19	***Positive***	No Data	Case	37	M	***13:01:01:01***	51:06:01:01	Yes
20	***Positive***	No Data	Case	54	M	***13:01:01:01***	40:06:01:02	Yes
21	***Positive***	Probable	Case	45	F	***13:01:01:01***	40:06:01:02	Yes
22	***Positive***	Probable	Case	28	F	***13:01:01:01***	15:01:01:01	Yes
23	***Positive***	Probable	Case	52	M	***13:01:01:01***	48:01:01:01	Yes
24	***Positive***	Probable	Case	43	F	***13:01:01:01***	52:01:01:01	Yes
25	***Positive***	Probable	Case	25	M	***13:01:01:01***	48:01:01:01	Yes
26	***Positive***	na	Independent	40	M	07:02:01:01	***13:01:01:01***	Yes
27	***Positive***	No Data	Case	18	F	***13:01:01:01***	35:03:01:01	Yes
28	***Positive***	Probable	Case	15	M	***13:01:01:01***	15:01:01:01	Yes
29	***Positive***	No Data	Case	18	F	***13:01:01:01***	18:01:01:02	Yes
30	***Positive***	Probable	Case	32	M	***13:01:01:01***	40:06:01:02	Yes
31	***Positive***	na	Independent	38	M	***13:01:01:01***	48:01:01:01	Yes
32	***Positive***	Probable	Case	37	F	***13:01:01:01***	35:01:01:02	Yes
33	***Positive***	No Data	Case	24	M	***13:01:01:01***	40:06:01:02	Yes
34	***Positive***	Probable	Case	24	M	***13:01:01:01***	44:03:02:01	Yes
35	***Positive***	na	Independent	33	F	***13:01:01:01***	15:01:01:01	Yes
36	***Positive***	No Data	Case	45	F	13:148	51:01:01:01	***No***
37	***Positive***	na	Independent	52	M	***13:01:01:01***	39:01:01:03	Yes
38	***Positive***	na	Control	42	F	***13:01:01:01***	56:01:01:03	Yes
39	***Positive***	na	Control	41	M	***13:01:01:01***	52:01:01:02	Yes
40	***Positive***	na	Control	20	F	***13:01:01:01***	52:01:01:01	Yes
41	***Positive***	na	Control	40	M	***13:01:01:01***	15:05:01:01	Yes
42	***Positive***	na	Control	40	M	***13:01:01:01***	15:32:01	Yes
43	Negative	na	Independent	84	M	15:01:01:01	44:03:02:01	Yes
44	***Positive***	na	Independent	38	F	***13:01:01:01***	48:01:01:01	Yes
45	***Positive***	na	Independent	76	M	***13:01:01:01***	38:02:01:01	Yes
46	Negative	na	Independent	52	F	07:02:01:01	44:03:02:01	Yes
47	***Positive***	na	Independent	27	M	***13:01:01:01***	15:05:01:01	Yes
48	Negative	na	Independent	24	M	15:25:01:01	18:01:01:02	Yes
49	Negative	na	Independent	30	F	15:25:01:02	58:01:01:01	Yes
50	***Positive***	na	Independent	30	M	***13:01:01:01***	52:01:01:01	Yes
51	Negative	na	Independent	NA	F	15:25:01:01	48:01:01:01	Yes
52	Negative	na	Independent	31	F	15:25:01:01	48:01:01:01	Yes
53	Negative	na	Independent	6	M	07:02:01:01	15:02:01:01	Yes
54	Negative	na	Independent	NA	F	44:03:02:01	44:03:02:01	Yes
55	Negative	na	Independent	70	M	37:01:01:01	48:01:01:01	Yes
56	Negative	na	Independent	70	F	35:01:01:02	35:01:01:02	Yes
57	Negative	na	Independent	28	M	15:25:01:01	58:01:01:01	Yes
58	***Positive***	na	Independent	28	M	***13:01:01:01***	48:01:01:01	Yes

*not applicable; ^#^Independent validation cohort; ^&^Data unavailable to classify DRESS category

### Differences in Demographics of DHS Cases with and without HLA-B*13:01

As 23.5% (8/34) DHS cases were negative for HLA-B*13:01 by both qPCR and NGS, we assessed potential differences between the clinical features of DHS cases with and without HLA-B*13:01. Dates of MDT start and final DHS diagnosis were available for twenty-four DHS cases (17 HLA-B*13:01 positive and 7 negative). The average duration between MDT start and DHS diagnosis for HLA-B*13:01 positive and negative DHS cases (36 days vs 44 days) was not significantly different (p = 0.31) (Fig 6). The two groups were further characterized based on sex, age, proportions with fever, skin rash and jaundice and ethnicities (Table 5). DRESS severity score is a probability score which categorizes drug-hypersensitive cases, based on a set of predefined clinical features occurring during hypersensitivity episodes, into probabilistic categories: Definite, Probable, Possible and Not DRESS. DRESS category was not considered for comparison as it was more probabilistic category rather than a severity category. Our analyses were based on available clinical data and not all cases had all relevant clinical/lab data available. Cases in both groups were found to be very similar in most of the demographic and clinical criteria except age. Despite a small sample size (26 vs 8 in allele positive and negative respectively), there was significant difference in median age (35.5 years vs 66 years, p = 0.0018).

**Table 5:** Clinical and demographic differences in HLA-B*13:01 positive and negative DHS cases. Comparison for DRESS categories was considered for analysis as the categories are probabilistic categories. Denominators depict the numbers of data available for analyses.

Details	HLA-B*13:01 (+)	HLA-B*13:01 (-)	p-value
**Sample size**	26	8	
**Males**	17/26	2/26	0.1
**Median Age**	35.5 years	66 years	**0.0018**
**Age Range**	15-72 years	33-78 years	na^#^
**Median Max BI**	1	0.5	0.87
**% with Fever^#^**	94% (15/16)	87.5% (7/8)	1
**% with Rashes**	100% (14/14)	87.5% (7/8)	0.33
**% Jaundice**	100% (17/17)	100% (8/8)	1
**Ethnicities**			
Brahmin/Chhetri	26.5% (9/26)	2 (25% (2/8)	0.16
Janajati	32.3% (11/26)	112.5% (1/8)	ns
Madhesi	11.8% (4/26)	37.5% (3/8)	ns
Tharu	3% (1/26)	25% (2/8)	ns
Newar	3% (1/26)	0% (0/8)	ns
**DRESS Category**			
Definite	0% (0/16)	3/8 (37.5%)	ns
Probable	93.4% (15/16)	5/8 (62.5%)	
Possible	6.3% (1/16)	0/8 (0%)	
Not DRESS	0% (0/16)	0/8 (0%)	

^**#**^Analyses were based on those with available clinical data.

# not applicable

**Fig 6:**
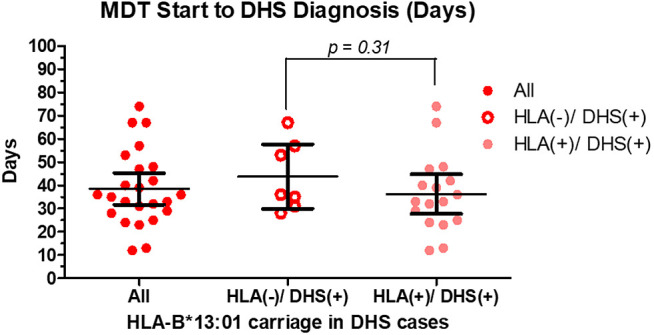
Duration between start of MDT to DHS diagnosis in HLA-B*13:01 positive and negative DHS cases. Red solid circles- All DHS cases; Red open circles- HLA-B*13:01 negative DHS cases; Pink circles- HLA-B*13:01 positive DHS cases.

## Discussion

The association of HLA-B*13:01 with DHS has been validated across multiple countries and ethnic groups; however, the magnitude of the association may vary in populations that have not yet been studied. Nepal remains one of the countries reporting high annual new leprosy case detection (10^th^ by number of cases reported for 2023) [45]. Nepal’s population composition is genetically diverse comprising both North Indian and South East-Asian linkages [50,61]. The magnitude of incidence of DHS was suggested to be associated with HLA-B*13:01 frequencies in regional populations [62]; and, according to the HLA Allele Net Database, the frequency of the allele is higher in Southeast Asian and Oceanian populations [42].

HLA typing, which is frequently used in the context of organ transplants, can be nonexistent or prohibitively expensive in low resource contexts. In Nepal, HLA typing is currently priced equivalent to $35-$100 USD per test; and the detailed report with HLA typing is unnecessary and requires extensive time beyond clinical relevance for leprosy treatment. In the present study in Nepal, we utilized a qPCR-based method which is comparatively less expensive and can be performed in any laboratory with basic qPCR facilities, such as for diagnostic SARS-CoV-19 qPCR. During the COVID-19 pandemic, qPCR laboratories grew in number, thereby developing or improving network of laboratories and expertise in some low resource contexts [63]. Thus, while many who diagnose leprosy globally may not necessarily have qPCR capacity in-house, access to private or government diagnostic qPCR laboratories may have improved.

The OR reported in our study was within the confidence intervals of previous meta-analyses: OR 42.69 with 95% CI: 42.7-63.0% [46] and OR 43.0 with 95% CI, 24.0-77.2 [47]. As most of the cases were confirmed DRESS, and many of our cases were retrospective in nature, we did not segregate our cases into SCAR classes: DRESS and SJS-TEN. Tangamornsuksan’s group (2018) [47] reported slightly higher OR for DRESS (OR 60.8) than SJS-TEN (OR 40.5). DHS is rarely reported from Americas but more frequently reported in South Asia and Southeast Asia [10,64]. This coincides with reported high prevalence of HLA-B*13:01 in Asian countries compared to elsewhere. Despite a statistically significant association between HLA-B*13:01 and DHS in our study, a small number of non-allergic HLA-carriers and allergic non-HLA-carriers were observed in our study and elsewhere [46,47]. This might hint the presence of other factors required for DHS occurrence and some other alternative alleles responsible for DHS, respectively. When comparing HLA-B*13:01 positive and negative DHS cases, we found DHS cases negative for the allele were significantly older than the allele positive DHS cases. Higher age has been repeatedly found to be associated with drug allergies [10,65,66].

Our study detected one HLA-B*13:148 positive DHS case that was negative for HLA-B*13:01 by NGS, potentially indicative of a possible non-HLA-B*13:01 HLA allele that could be related to DHS. HLA-B*13:148 has an identical amino acid sequence in the peptide binding groove compared to HLA-B*13:01. As this allele was detected in a clinically confirmed DHS case, and the allele (HLA-B*13:148) sequence differed by only one SNP compared to HLA-B*13:01, it was considered that the qPCR was still useful to help clinicians to make treatment decisions. Professor Wang’s group (2013) also found a non-HLA-B*13:01 allele HLA-B*13:13, which has also identical amino acids in the groove as HLA-B*13:01, in two out of their 20 DHS cases [38]. It has been reported that the drug dapsone binds/docks to F pocket in the peptide binding grove [23, 25]. Small variation in the amino acids lining the F pocket could render the binding inefficient. This docking of dapsone probably alters the repertoire of peptides binding to the class I MHC HLA-B*13:01 [16,22] eliciting T cell stimulation to the new non-self peptides. Changes in peptide repertoire or even changes in the 3-dimentional conformation of the peptides have been shown to elicit T cell stimulations during drug hypersensitivity [67, 68]. Multiple drugs have also been shown, *in silico*, to bind multiple HLAs [69]. As more than ten thousand class I HLA alleles are found, there is plausible chance that dapsone could bind to other HLAs at suitable sites to change the peptide repertoire.

Regarding ethnicity, the study results should be interpreted cautiously as Nepal is a diverse country with 142 recognized ethnicities [59]. Approximately 27% of the Nepalese population is comprised of Brahmin/Chhetris, 21% of Madhesis (including Madhesi Dalits) and 35% of Janajatis [59]. Both Madhesi and Janajati populations (~56% of Nepalese) are highly heterogenous genetically. While the association shown in our study do not correspond to a particular Nepalese ethnicity, presumptive differences in HLA-B*13:01 occurrence between ethnicities are unlikely to be practically significant in clinical settings; and preventative screening should be part of personalized medicine. Across ethnicities, the study validates the established association with high confidence and endorses the applicability of preventative screening for the allele before dapsone is initiated as part of MDT.

As in many limited resource settings, suspect DHS cases in Nepal often travel long and difficult distances to be finally diagnosed by experts as often occurs with leprosy complication cases [70]. As many of the newly diagnosed leprosy cases return home and continue the MDT at local government health institutions, eruption of DHS symptoms may be difficult to be diagnosed and managed locally. Due to the economic hardships common among many leprosy patients in Nepal and elsewhere, a more affordable preventative point-of-care test for HLA-related risk for DHS at diagnosing centers would be ideal.

Unlike diseases caused by specific microbial etiological agents, HLA is a human gene, and the gene copy number in a 100 µl blood sample (average ~ 7.5K WBC/µl) is high, making it highly suitable for nucleic acid amplification-based tests. Studies using the Loop-mediated isothermal amplification (LAMP) method to detect specific HLA-related risk to prevent drug hypersensitivity have been published [71, 72]. Moreover, protocols have been optimized to use whole blood directly to detect presence of parasites in human blood [73–75], or even the specific HLA (HLA-A*31:01) causing (carbamazepine-induced) hypersensitivity [76]. A simple PCR-based line probe assay to monitor antimicrobial drug resistance through detection of mutation/SNP of *Mycobacterium leprae* drug-target genes has already been recommended by the WHO [77,78]. Similar field-friendly and readily interpretable assays could improve the applicability of the genetic test in leprosy patients before administration of MDT. While the prevalence of this HLA is variable in different parts of the world, the assay could still be vital in saving significant morbidity and mortality in regions where both the allele prevalence and leprosy caseloads are high [62].

**Fig 7:**
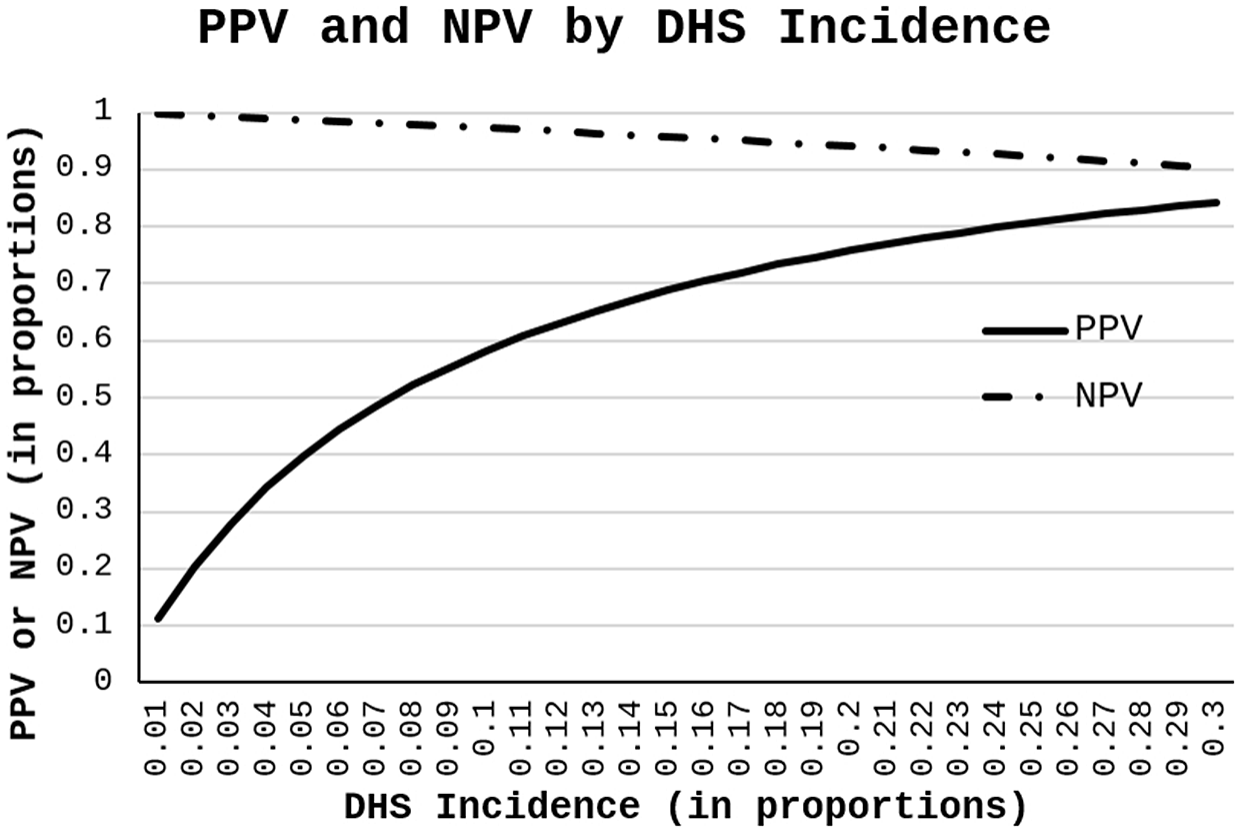
PPV (solid line) and NPV (dashed line) values based on DHS incidence with sensitivity of 0.765 and specificity of 0.939. The graph shows that with the same sensitivity and specificity, the PPV and NPV change based on background population DHS incidence.

The usability of an assay also depends upon the positive predictive value (PPV) of the test, which is the proportion of DHS cases among the test positive cases. The PPV, in turn, depends upon the incidence rate of DHS (Fig 7). DHS incidence varies widely in different countries. The worldwide incidence was estimated to be around 1.4% in a systematic review [10] and a similar percentage (1.5%) was reported in China [62]. However, some countries report higher incidences. Indonesia reported ~10% [41] DHS incidence, and Nepal has a previously reported incidence between 2–3% [44,79,80]). The PPV increases as incidence increases, as shown in Fig 7 drawn with sensitivity and specificity values of the present study. In our case, considering the Nepalese DHS incidence to be ~ 2.5%, the present sensitivity (76.5%) and specificity (93.9%), predicts the DHS with PPV of 24.3% (95% CI: 11.9% to 43.4%) and NPV of 99.4% (95% CI: 98.8% to 99.7%). Positive and Negative Likelihood Ratios (LRs) are other statistic metrics used to gauge a test. Diagnostic tests with positive LR (12.5 in calculated in the present study) higher than 10 and Negative LR (0.25 calculated) lower than 0.1 are generally considered to provide strong evidences for ruling in and out a disease respectively [81]. High positive LR suggests high applicability for HLA-B*13:01 screening test to prevent DHS. Additionally, the test with a moderate negative LR value [82, 83] supported by a high NPV (99.4% at ~2.5% DHS incidence in Nepali population) can provide valuable information for clinicians to decide whether to initiate dapsone (as part of MDT) treatment. The arithmetic sensitivity and specificity of HLA-B*13:01 positivity in DHS cases, based on all published data until present, was 85.4% and 88.0% respectively (Table 6). Given a significant incidence in Southeast Asian countries of DHS (1–10%), it is imperative to include screening of the allele before administration of MDT in leprosy cases, especially in these regions. As leprosy is more prevalent in economically restrained populations [84], the screening test can prevent the high mortality rate (~10%, [10]) and catastrophic treatment costs. Some other studies have already reported qPCR-based methodology for HLA-B*13:01 without NGS validation and one with NGS validation [85–87].

**Table 6:** Calculation of arithmetic sensitivity and specificity with all available published data and data from this study. Individual articles are cited in the text.

Study	Country	*DHS Cases*		*Dapsone-Tolerants*	
		HLA(+)	Total (n)	HLA(+)	Total (n)
[38]	China	18	20	7	102
[39]	China	65	76	148	1034
[30]	Thailand	12	15	2	29
[20]	Taiwan	7	8	*Not available*	
[46]	Korea	6	7	1	8
[40]	India	7	8	9	113
[41]	Indonesia	31	34	2	52
[24]	Thailand	13	16	4	40
Menaldi 2025	Indonesia	8	8	3	53
This study, 2026	Nepal	26	34	5	82
**Total**		**193**	**226**	**181**	**1513**
Sensitivity			**85.4%**		
Specificity					**88.0%**

With regard to limitations, the sample size for cases was modest in the present study. Based on the study duration (2 years) and estimated incidence of DHS in Nepal (~2.5%), it would have been very difficult to enroll enough sample population given the annual new case detection of 150–200 cases at the study site. Additionally, due to implementation of HLA-B*13:01 screening during the study period, the incidence of DHS in new patients (HLA-B*13:01 positive new cases not prescribed dapsone) at the study site decreased significantly (manuscript under preparation); and thus, the present study relied on those DHS cases who were previously treated at the hospital site and visited the hospital during the study period for leprosy-related problems, and those who were referred from other centers in Nepal. Despite our modest sample size, based on the power calculation criteria, the sample size has sufficient power to estimate the OR.

According to the design of the present study, only non-fatal DHS cases could be enrolled in the study. As fatal cases account up to 10% of the DHS cases, the data could be more accurate if we had also enrolled cases who died due to DHS. This limitation also existed in other studies where primarily retrospective cases were enrolled [38, 39]. The impact of homozygosity of HLA-B*13:01 in severity of DHS episodes was not studied, as there was only one homozygous HLA-B*13:01 positive case in our study. The qPCR methodology used in our study was more feasible than the methods used in other studies (NGS, etc.), although a basic molecular biology setting is still necessary to perform qPCR. More field-friendly methodologies [72] could improve the applicability of the test. A considerable proportion (23.5%) of the cases in the present study were HLA-B*13:01 negative, and thus the role of alternative HLA alleles [49] in DHS causation is plausible. Further studies in identifying potential new alleles in DHS causation could help further reduce DHS incidence. Nepal is a multi-ethnic country, and the association of HLA-B*13:01 with DHS is affected by the genetic background of patient population at the study site. While the present study analyzed samples from people living in different parts of Nepal, the largest group was in the districts nearest the hospital; and, thus, it may not have captured samples more reflective of the Southern Terai region where the patient population may be genetically different.

Implementation of preventative HLA-B*13:01 qPCR in Chinese [88] and studies in Indonesian [41,89] and Nepalese populations (manuscript in progress) have demonstrated drastic reductions to near zero cases in DHS. Indonesia is the third highest reporting nation for leprosy with around 16,000 new cases detected annually. An incredible 91% of the tested Indonesian DHS population was shown to be positive for HLA-B*13:01 [41]; and it was announced that, since late 2025, HLA-B*13:01 qPCR preventive screening would become available for all new leprosy cases in Indonesia [90]. As more HLA-B*13:01 qPCR studies or implementations are performed across diverse and international populations evidence may broaden, the sensitivity and specificity may become better defined, and, ideally, DHS will be prevented. Two out of three major countries (India, Brazil and Indonesia) contributing to global leprosy caseloads [45] have already reported, though in limited ethnic backgrounds, the association between their DHS cases and HLA-B*13:01. Though DHS is rarely reported in Brazil and African countries [10,64], data from Southeast Asian countries, including ours, support the utility of HLA-B*13:01 pre-screening before MDT prescription to prevent the consequences of DHS in the region. Approximately 72% of global leprosy caseloads are present in Southeast Asian countries [45]. Considering a modest ~1.4% [10] incidence of DHS in Southeast Asia (with 131,425 new cases detected in 2023), introduction of HLA-B*13:01 pre-screening may prevent serious morbidities due to DHS in approximately 1,800 newly diagnosed leprosy cases each year. Implementation of HLA-B*13:01 pre-screening may result in up to three-quarters (PPV 24.3%) of the HLA-B*13:01-positive cases in Nepal (proportions may vary in different settings) being prescribed alternative leprosy treatments [88,91], whose efficacy remains insufficiently studied. Recommendations for HLA-B*13:01 pre-screening should be complemented with research for better alternative treatments [92–94]. A small proportion (~0.5%, NPV 99.4%) of leprosy cases in our study (proportions may vary in different settings) are still at risk of developing DHS]). Further studies to identify the additional risk factors in the allergic non-carrier (of the risk alleles) population, for example, the contribution of multiple HLA alleles [95], need to be undertaken to further reduce the incidence of DHS. Our study complements existing evidence on the need for HLA-B*13:01 screening and provides reference data for national and international stakeholders seeking to improve the safety and adherence to leprosy treatment.

## Supporting information

S1 File Demographic, clinical and HLA typing (by qPCR) results of study participants. (XLSX)

S2 File Consolidated Meta analysis with PRISMA. (DOCX)

S1 Table Characteristics of included studies in the meta-analysis. (XLSX)

S2 Table Newcastle-Ottawa Scale Risk of Bias Assessment for included studies in meta-analysis. (XLSX)

S3 Table Summary statistics for individual studies included in meta-analysis. (XLSX)

S1 Fig PRISMA Flow-chart for meta-analysis. (TIF)

S2 Fig Forest plot of meta-analysis of 8 case-control studies. (TIF)

S3 Fig Contour-enhanced funnel plot (white p > 0.1, dark grey p < 0.1, gray p < 0.05, light grey p < 0.01). (TIF)

S1 Strobe checklist STROBE checklist. (DOCX)
