# Supplementary material for: Evaluating the risk of dapsone hypersensitivity syndrome in Nepalese Leprosy Patients via HLA-B* 13:01 screening using real-time PCR and comparative meta-analysis of international data
Source: PLoS Negl Trop Dis. 2026 Aug 13;20(8):e0014568. doi: 10.1371/journal.pntd.0014568 (PMC13472375; doi:10.1371/journal.pntd.0014568)
Supplement: Supplementary file 1 [file pntd.0014568.s002.docx]

**INTRODUCTION**

**Item 1. Title: Identify the report as a systematic review.**

Meta-analysis is sub-part of an article with an appropriate title

**Item 2. Abstract: See the PRISMA 2020 for Abstracts checklist.**

Meta-analysis is mentioned in abstract

**Item 3. Rationale: Describe the rationale for the review in the context of existing knowledge.**

Multiple studies have shown association between HLA-B*13:01 and Dapsone Hypersensitivity Syndrome (DHS) in their respective ethnic populations (Chiramel et al., 2019; Krismawati et al., 2020; Park et al., 2020; Satapornpong et al., 2021b, 2021a; Tempark et al., 2017; H. Wang et al., 2013; Zhang et al., 2013). Dapsone is approved for use in leprosy and dermatitis herpetiformis (Wolf & Orni-Wasserlauf, 2000; Wozel & Blasum, 2013; Zuidema et al., 1986). Dapsone is provided as a part of multidrug therapy (MDT: dapsone, rifampicin and clofazimine) recommended by World Health Organization (WHO) for leprosy affected people around the world (WHO, 1982, 2018). Improved understanding on the impact of HLA-B*13:01 carriage on occurrence of DHS can valuable clues for shift in policies regarding modification of MDT (N. Wang et al., 2017).

**Item 4. Objectives: Provide an explicit statement of the objective(s) or question(s) the review addresses.**

The meta-analysis was aimed to estimate the pooled odds ratio for occurrence of DHS in HLA-B*13:01 positive cases compared to HLA-B*13:01 negative cases.

**METHODS**

**Eligibility criteria: Item 5. Specify the inclusion and exclusion criteria for the review and how studies were grouped for the syntheses.**

**Inclusion criteria:**

- Retrospective or prospective case control studies comparing DHS occurrence in HLA-B*13:01 positive cases.
- Daily dosages of >50 mg dapsone in adults of ≥18 years.
- Any disease including leprosy where dapsone is prescribed for at least 14 days
- DHS should be diagnosed according to Richardus and Smith criteria (Richardus & Smith, 1989), RegiSCAR DRESS (Kardaun et al., 2013) criteria or Japanese DiHS criteria or any standard regional criteria (Brüggen et al., 2024; Shiohara & Mizukawa, 2019)
- Controls should have taken at least 8 weeks without any adverse events to rule out any DHS symptoms (Agrawal & Agarwalla, 2005; Kosseifi et al., 2006; Lorenz et al., 2012; Richardus & Smith, 1989; N. Wang et al., 2017)
- Analyzed for severe cutaneous adverse events (SCARs) together (DRESS and/or SJS/TEN)

**Exclusion criteria:**

- Case report or case series
- DHS occurrence in HLA-B*13:01 positive patients compared against population rather than controls taking dapsone.
- Studies before 2013, as the HLA-B*13:01 and daposne hypersensitivity association was reported in 2013 (H. Wang et al., 2013; Zhang et al., 2013).
- Non severe cutaneous adverse events (SCARs) including Maculopapular exanthema (MPE), Fixed Drug Eruptions (FDE), urticarial,	etc. (Brockow et al., 2015; Roujeau, 2005).

**Information sources: Item 6. Specify all databases, registers, websites, organisations, reference lists and other sources searched or consulted to identify studies. Specify the date when each source was last searched or consulted.**

PubMed and Embase databases were searched up to second week of March 2026. Only English language articles were used. Articles not found in PubMed and Embase but referenced in articles found there would also become eligible if the data were published in any peer-reviewed journal.

**Search strategy: Item 7. Present the full search strategies for all databases, registers and websites, including any filters and limits used.**

PubMed:

A: ((dapsone hypersensitivity) OR (sulfone allergy)) OR (dapsone allergy) = 6290 results

B: human leukocyte antigen = 159,860 results

A AND B: 104 results

Embase:

A: 'dapsone hypersensitivity' OR (('dapsone'/exp OR dapsone) AND ('hypersensitivity'/exp OR hypersensitivity)) OR 'sulfone allergy' OR (('sulfone'/exp OR sulfone) AND ('allergy'/exp OR allergy)) OR 'dapsone allergy' OR (('dapsone'/exp OR dapsone) AND ('allergy'/exp OR allergy)) = 7473 results

B: 'hla antigen'/exp OR 'hla antigen' = 140,694

A AND B = 180 results

Google:

Google search engine was utilized to search for any missing studies utilizing similar terms.

**Selection process: Item 8. Specify the methods used to decide whether a study met the inclusion criteria of the review, including how many reviewers screened each record and each report retrieved, whether they worked independently, and if applicable, details of automation tools used in the process.**

Articles were searched and decided to be included in meta-analysis by first author (DRR). The correctness of the process were reviewed by two senior authors (PS and DAH). No automation tools were used.

**Data collection process: Item 9. Specify the methods used to collect data from reports, including how many reviewers collected data from each report, whether they worked independently, any processes for obtaining or confirming data from study investigators, and if applicable, details of automation tools used in the process.**

Data collection by first author and reviewed by two senior authors.

**Data items:**

- **Item 10a. List and define all outcomes for which data were sought. Specify whether all results that were compatible with each outcome domain in each study were sought (e.g. for all measures, time points, analyses), and if not, the methods used to decide which results to collect.**

Outcomes included sample sizes for cases and controls and number of HLA-B*13:01 positivity in cases and controls irrespective of age, sex, ethnicity, etc.

- **Item 10b. List and define all other variables for which data were sought (e.g. participant and intervention characteristics, funding sources). Describe any assumptions made about any missing or unclear information.**

If the country of origin of cases and control were same, then it was assumed that ethnicity and geographical origin was matched. As this is a genetic study, the age of participant and sex (autosomal gene) did not impact overall estimation of HLA prevalence, thus age and sex differences did not impact selection. Different methods for HLA determination were used. Thus all recognized methodologies were considered satisfactory. PCR if used need to be cross-validated. All demographic variables required to be matched statistically.

**Study risk of bias assessment Item 11. Specify the methods used to assess risk of bias in the included studies, including details of the tool(s) used, how many reviewers assessed each study and whether they worked independently, and if applicable, details of automation tools used in the process.**

Newcastle Ottawa Scale (NOS) was used to study the risk of bias (Gualdi-Russo & Zaccagni, 2026; Wells et al., 2000). There are 3 domains (Selection, Comparability and Exposure). The questions, 4 in Selection, 2 in Comparability and 3 in Exposure were used as they were. For Comparability, Ethnicity and Timing of enrolments of case and controls were taken as two important factors. If any study did matching of ethnicity and timing, it was considered that the study complied with the criteria. But if it did not clearly mention matching, then the study was considered to have failed to comply with that criteria. NOS was implemented by one author (DRR) and 2 senior authors (PS and DAH) reviewed the process.

**Effect measures Item 12. Specify for each outcome the effect measure(s) (e.g. risk ratio, mean difference) used in the synthesis or presentation of results.**

Odd ratio was calculated for cases vs control. HLA-B*13:01 positivity was considered exposure. Two field or 4 digit resolution was required for HLA typing. For example, HLA-B*13:01 was enough and HLA-B*13:01:01 or HLA-B*13:01:01:01 was not required. Number of cases and control and number of HLA-B*13:01 positivity in cases and controls were used to calculate pooled OR in the meta-analysis. If any study did logistic regression to calculate OR, the respective OR were not used and raw case/control data were utilized. If only population control (without data on AE for dapsone intake) was used, the study was excluded from analysis.

**Synthesis methods: Item 13a. Describe the processes used to decide which studies were eligible for each synthesis (e.g. tabulating the study intervention characteristics and comparing against the planned groups for each synthesis (item #5)).**

As this was a consolidated meta-analysis with the only objective to summarize association between HLA-B*13:01 and dapsone hypersensitivity, no other sub-group analyses were performed. Articles were selected based on inclusion and exclusion criteria mentioned in item 5. All case control studies investigating the association between HLA-B*13:01 and dapsone hypersensitivity were eligible for synthesis given the complied with exclusion and inclusion criteria.

**Item 13b. Describe any methods required to prepare the data for presentation or synthesis, such as handling of missing summary statistics, or data conversions.**

Data were available for all included studies. One study (Chiramel et al., 2019) did not explicitly define Controls but were still included in the study, given that authors understood the definitions of controls in a leprosy endemic country where dapsone hypersensitivity is frequently reported.

**Item 13c. Describe any methods used to tabulate or visually display results of individual studies and syntheses.**

R statistical software (meta package) was utilized to perform meta-analysis, draw forest and funnel plots.

**Item 13d. Describe any methods used to synthesize results and provide a rationale for the choice(s). If meta-analysis was performed, describe the model(s), method(s) to identify the presence and extent of statistical heterogeneity, and software package(s) used.**

“meta” package in R statistical software with RStudio 2023.12.0 Build 369 was utilized for meta-analysis. Inverse-variance method for Fixed/Common effect and Restricted Maximum Likelihood for Random effects were used. The heterogeneity was tested with I-squared and Tau-squared tests.

**Item 13e. Describe any methods used to explore possible causes of heterogeneity among study results (e.g. subgroup analysis, meta-regression).**

No sub-group analyses were made.

**Item 13f. Describe any sensitivity analyses conducted to assess robustness of the synthesized results.**

No sensitivity analyses were tested. Based on simple objective to identify the pooled estimate, sensitivity analysis was not the scope. The included studies found significant association in same direction.

**Reporting bias assessment: Item 14. Describe any methods used to assess risk of bias due to missing results in a synthesis (arising from reporting biases).**

Publication bias was calculated using Begg’s Rank correlation test in R (Begg & Mazumdar, 1994). Egger test was not utilized as this is suggested only for meta-analyses with 10 or more individual studies. So, Begg’s test with rank correlation was used using minimum of 8 studies.

**Certainty assessment: Item 15. Describe any methods used to assess certainty (or confidence) in the body of evidence for an outcome.**

Additional certainty of evidence was not assessed. Risk of Bias was assessed using New Castle Ottawa scale. The Newcastle-Ottawa Scale scores were categorized in to Good, Fair and Poor quality studies based on Agency for Healthcare Research and Quality (AHRQ) method (Shamsrizi et al., 2020).

Good quality: 3 or 4 stars in selection domain AND 1 or 2 stars in comparability domain AND 2

or 3 stars in outcome/exposure domain

Fair quality: 2 stars in selection domain AND 1 or 2 stars in comparability domain AND 2 or 3

stars in outcome/exposure domain

Poor quality: 0 or 1 star in selection domain OR 0 stars in comparability domain OR 0 or 1 stars

in outcome/exposure domain

**RESULTS**

**Study selection: Item 16a. Describe the results of the search and selection process, from the number of records identified in the search to the number of studies included in the review, ideally using a flow diagram.**

PubMed and Embase databases were used. Supplementary search were made in Google search engine to identify and studies not reported by PubMed and Embase. Please see Methods for search terms. 284 studies were retrieved from both databases (PubMed 104 and Embase 180). 29 were duplicates, 83 were published before 2013, and 162 were review articles or article not under our objectives. One result was obtained from Google. Eight studies were used for synthesis (S1 Fig).

S1 Fig: PRISMA Flow-chart for meta-analysis.

**Item 16b. Cite studies that might appear to meet the inclusion criteria, but which were excluded, and explain why they were excluded.**

Ten results were short-listed from the two databases. Two were removed because they were conference articles and no detailed report were available, both from Thai populations.(Sukasem, 2019; Sukasem et al., 2017). Further one result (Chen et al., 2018) was not included in the study because they compared their case data with population control and no clinically dapsone-tolerant controls were used.

**Study characteristics: Item 17. Cite each included study and present its characteristics.**

The characteristics of the study are reported here in the tabular format ( S1 Table). Eight studies were included in this trial. Two were from China (H. Wang et al., 2013; Zhang et al., 2013), 2 from Indonesia (Krismawati et al., 2020; Menaldi et al., 2025), 2 from Thailand (Satapornpong et al., 2021b; Tempark et al., 2017), one from India (Chiramel et al., 2019)and one from South Korea(Park et al., 2020).

S1 Table: Characteristics of included studies in the meta-analysis

**Risk of bias in studies: Item 18. Present assessments of risk of bias for each included study.**

Seven out of 8 studies ranked best category (GOOD quality) and one ranked moderate category (FAIR quality). None ranked in lowest category of POOR quality (S2 Table). Ethnicity reporting was not present in many. Though the countries may be highly homogenous (Korea, Thailand, Taiwan), lack of any statement about the ethnicity matching were considered violation of the Newcastle Ottawa Scale(Gualdi-Russo & Zaccagni, 2026; Wells et al., 2000).

S2 Table: Newcastle-Ottawa Scale Risk of Bias Assessment for included studies in meta-analysis

**Results of individual studies: Item 19. For all outcomes, present, for each study: (a) summary statistics for each group (where appropriate) and (b) an effect estimate and its precision (e.g. confidence/credible interval), ideally using structured tables or plots.**

The odds ratio for individual studies ranged from 39 (Satapornpong et al., 2021a) to 258.3 (Krismawati et al., 2020). See S3 Table.

S3 Table: Summary statistics for individual studies included in meta-analysis

**Results of syntheses: Item 20a. For each synthesis, briefly summarise the characteristics and risk of bias among contributing studies.**

The first HLA and DHS association study in 2013 was, by far, the biggest study in terms of sample size (Zhang et al., 2013). The study was only one which used microarray technique first to identify the signature SNP, later validated by sequencing. All other studies used sequence specific probes, qPCR or sequencing. Sequencing were both Next-Generation Sequencing and capillary (Sanger) sequencing method. The studies using the capillary sequencing method used software to differentiate the sequencing data into the two HLA-B alleles in humans.

Risk of bias have been tabulated above (S2 Table). One study (Park et al., 2020) which was categorized as having a “Fair” Risk of Bias had no case representativeness, no appropriate control selection and no ethnicity comparison. Total 4 out of 8 studies didn’t clearly mentioned ethnicity matching (Chiramel et al., 2019; Park et al., 2020; Satapornpong et al., 2021b; Tempark et al., 2017). One Indonesian study (Krismawati et al., 2020) didn’t provide data for enrolment time. Control definition was not clear for 2 studies (Chiramel et al., 2019; H. Wang et al., 2013).

**Item 20b. Present results of all statistical syntheses conducted. If meta-analysis was done, present for each the summary estimate and its precision (e.g. confidence/credible interval) and measures of statistical heterogeneity. If comparing groups, describe the direction of the effect.**

OR by Random and Fixed (Common) Effect Models were 61.86 (95% CI 32.60 - 117.4, p < 0.0001) and 52.54 (95% CI 31.90 - 86.52, p < 0.0001) respectively (S2 Fig). All tests of heterogeneity showed lack of heterogeneity (tau^2 = 0.1582 (95% CI: 0.0000 - 1.6349); I^2 = 0.0% (95% CI: 0.0%; 67.6%); Chocran’s Q 6.52, df 7, p-value 0.4808). The included studies reported in same directions.

S2 Fig: Forest plot of meta-analysis of 8 case-control studies

**Item 20c. Present results of all investigations of possible causes of heterogeneity among study results.**

Heterogeneity was minimal. No sub-analyses were performed.

**Item 20d. Present results of all sensitivity analyses conducted to assess the robustness of the synthesized results.**

All the individual studies reported effect sizes in same direction. No sub-group analyses were performed due to the scope of this study. The scope was just to identify a pooled effect size of all published studies. Please see the text in main article.

**Reporting biases: Item 21. Present assessments of risk of bias due to missing results (arising from reporting biases) for each synthesis assessed.**

Rank correlation test (Begg & Mazumdar, 1994) showed no publication bias (z = 0.99, p-value = 0.3223) (S3 Fig). Visual screening of the contour-enhanced funnel plot (white p>0.1, dark grey p<0.1, gray p<0.05, light grey p<0.01) showed that the studies were equally distributed around the central line and all fall in highly significant areas. As the enrolled studies showed high association between HLA-B*13:01 and DHS.

S3 Fig: Contour-enhanced funnel plot (white p>0.1, dark grey p<0.1, gray p<0.05, light grey p<0.01).

**Certainty of evidence: Item 22. Present assessments of certainty (or confidence) in the body of evidence for each outcome assessed.**

Leprosy is endemic in few countries around the world. The problem of DHS is not present in all endemic countries or areas, perhaps due to differential prevalence of HLA-B*13:01. We attempted to identify all case-control studies that studied the association between these two factors. This study showed high association and very low heterogeneity. The confidence interval of the pooled OR is 61.86 (95% CI 32.60 - 117.4, Random effects) is wide but the lower limit is safely at satisfactory level. Thus we assume the association is significant across different parts of the world. No specific analyses were performed for certainty of evidence.
