# Supplementary material for: Evaluating the risk of dapsone hypersensitivity syndrome in Nepalese Leprosy Patients via HLA-B* 13:01 screening using real-time PCR and comparative meta-analysis of international data
Source: PLoS Negl Trop Dis. 2026 Aug 13;20(8):e0014568. doi: 10.1371/journal.pntd.0014568 (PMC13472375; doi:10.1371/journal.pntd.0014568)
Supplement: Supplementary file 4 [file pntd.0014568.s012.docx]

Dear Editor and Reviewers

Thank you for valuable comments in making the manuscript more improved. We have tried to answer all comments and make corresponding changes.

PNTD-D-26-00056R1

Evaluating the Risk of Dapsone Hypersensitivity Syndrome in Nepalese Leprosy Patients via HLA-B*13:01 Screening Using Real-Time PCR and Comparative Meta-Analysis of International Data

Please find the answers highlighted in yellow.

We would like to kindly remind that the corresponding authors of this manuscript are Dr. Deanna A Hagge: deanna@zeroleprosypathfinder.com; Dr. Mahesh Shah: drmahesh_shah@yahoo.com, maheshs@tlmnepal.org. It was depicted in the submitted manuscript from the beginning. Mr. Divya, first author, was responsible for submission process.

Thank you

Authors

Response to Reviewers

**Reviewers' comments:**

Reviewer's Responses to Questions

**Key Review Criteria Required for Acceptance?**


As you describe the new analyses required for acceptance, please consider the following:


**Methods**

-Are the objectives of the study clearly articulated with a clear testable hypothesis stated?

-Is the study design appropriate to address the stated objectives?

-Is the population clearly described and appropriate for the hypothesis being tested?

-Is the sample size sufficient to ensure adequate power to address the hypothesis being tested?

-Were correct statistical analysis used to support conclusions?

-Are there concerns about ethical or regulatory requirements being met?

Reviewer #2: The case–control design, qPCR-with-NGS validation, and analytic plan are appropriate for the pharmacogenetic question and were substantially clarified in this revision. One methodological point requires attention: the effect-estimate computation should be stated explicitly and applied consistently. The odds ratio appears as 50.1 (95% CI 15.0–166.6) in the text but 50.05 (95% CI 14.5–142.6) in Figure 4, and sensitivity is reported as both 77% and 76.5%. These differences are small but indicate more than one calculation method (e.g. Fisher exact vs logit/Woolf intervals). Please specify the single method used and propagate one value set throughout the manuscript and figures.

Answer: Apologies for the inconsistencies. Now corrected.

Reviewer #3: -Are the objectives of the study clearly articulated with a clear testable hypothesis stated? yes
-Is the study design appropriate to address the stated objectives? yes
-Is the population clearly described and appropriate for the hypothesis being tested? yes, although small population
-Is the sample size sufficient to ensure adequate power to address the hypothesis being tested? small population
-Were correct statistical analysis used to support conclusions? yes
-Are there concerns about ethical or regulatory requirements being met? No

Answer: Thank you. We had previously improved our discussions for sample size in Discussion paragraph 9.

**Results**


-Does the analysis presented match the analysis plan?

-Are the results clearly and completely presented?

-Are the figures (Tables, Images) of sufficient quality for clarity?

Reviewer #2: The Results are clearly presented overall, but several quantitative inconsistencies should be corrected before the central findings can be interpreted unambiguously:

Table 6 column labelling is internally impossible as printed. The second column ("HLA(-)") holds group totals rather than allele-negative counts — the present study row lists 34 and 82 under "HLA(-)" when the true negatives are 8 and 77. The pooled estimates are arithmetically correct (sensitivity 193/226 = 85.4%; specificity 1161/1312 = 88.5%, with 1161 = 1312−151), so the underlying calculation holds, but the table should be relabelled "Total (n)" or repopulated with true HLA-negative counts.
The Zhang 2013 denominators disagree between Figure 5 (controls 148/2064) and Table 6 (controls 119/833). Because this is the largest contributing study, the discrepancy materially affects the pooled estimate. Please reconcile the extraction across the forest plot, table, and text.
"Chen et al. 2018" (Table 6) and "WT et al. 2018" (forest plot) appear to be the same Taiwan paper (JID 138(7):1546–54). Please confirm it is not double-counted in the pooled sensitivity and merge the reference.
Confidence intervals are inconsistent across the manuscript: PPV 1.9–43.4 (Results) vs 11.9–43.4 (Discussion); NPV 98.8–99.6 (Results) vs 8.8–99.7 (Discussion, an apparent typo). These should be harmonised.
Table 5 subgroup denominators are uneven and imply unreported missingness — fever 15/16, rashes 14/14, jaundice 17/17 against group n = 26/8 — and "Probable 93.4%" does not match a /16 or /26 denominator. Please report explicit denominators and missingness per row.

Answer: Apologies for the typos. This has been harmonized across the manuscript and supplementary materials for meta-analyses. Sensitivity, specificity and predictive values are now presented with three digits (one digit after decimal) across the manuscript.

Table 6 corrected with new Zhang et al 2013 data. Corresponding changes made for meta-analyses. Chen et al 2018 data did not include controls in their study, thus, it was not included in the meta-analysis. It was not there from our initial submission. For arithmetic calculation (Table 6) of sensitivity and specificity in Discussion, it was included for Case data. WT et al 2018 and Chen et al 2018 are same. WT et al 2018 now replaced with Chen et al 2018.

Correcting the Zhang et al data led to slight changes summary OR (separate Random Effects and Common Effects reported now) for meta-analysis. The results (reported with 95% confidence intervals) do not significantly change compared to previous submission. Bias estimate was not significant. Forest plot (figure 5) replaced with the new estimation (and funnel plot in meta-analysis supplementary material was also replaced with new one).

For table 5, as mentioned in Results (second paragraph), nine and ten DHS cases did not have enough data for the application of the Richardus and Smith, and the RegiSCAR criteria respectively. Thus denominators for calculation were based on available data. As provided in supplementary material (please see Richardus and Smith criteria columns), fever, skin rash and jaundice data were available for 16, 14 and 17 participants in HLA-B*13:01-positive DHS cases (n = 26), and for all 8 in HLA-B*13:01-negative DHS case group (n = 8). DRESS criteria score was available for 16 of total 26 cases for HLA-B*13:01-positive DHS case group, and all 8 of 8 in HLA-B*13:01-negative DHS case group. Now all calculations in table show denominators. Data availability (as denominators) now mentioned in figure caption.

Reviewer #3: -Does the analysis presented match the analysis plan? yes
-Are the results clearly and completely presented? yes
-Are the figures (Tables, Images) of sufficient quality for clarity? Yes

Answer: Thank you.

**Conclusions**


-Are the conclusions supported by the data presented?

-Are the limitations of analysis clearly described?

-Do the authors discuss how these data can be helpful to advance our understanding of the topic under study?

-Is public health relevance addressed?

Reviewer #2: The conclusions are supported and the public-health relevance (pre-treatment screening to prevent a high-morbidity, ~10%-mortality reaction) is well-aligned with the journal. One interpretive correction: the manuscript states a negative likelihood ratio <0.1 provides strong rule-out evidence, then reports NLR = 0.25, which does not meet that threshold. As written it implies the test qualifies. The rule-out argument should rest on the prevalence-dependent NPV (99.4% at 2.5% incidence), not the NLR; please revise the wording so 0.25 is described as moderate.

Answer: Thank you. Corresponding changes now made.

Reviewer #3: -Are the conclusions supported by the data presented? needs a more explicit conclusion with recommendations and implications
-Are the limitations of analysis clearly described? yes
-Do the authors discuss how these data can be helpful to advance our understanding of the topic under study? no
-Is public health relevance addressed? Yes

Answer: Thank you. Additional sentences including references added at the end of Discussions.

**Editorial and Data Presentation Modifications?**


Use this section for editorial suggestions as well as relatively minor modifications of existing data that would enhance clarity. If the only modifications needed are minor and/or editorial, you may wish to recommend “Minor Revision” or “Accept”.

Reviewer #2: Minor, all tied to the consistency issues above: regenerate Tables 5 and 6 and Figures 4–5 from the cleaned dataset with row/column totals that sum correctly and self-contained captions; standardise the single OR/sensitivity value set throughout; and merge the duplicate Chen/WT 2018 reference. These are correctable as part of a minor revision.

Answer: All corrected now. Thank you.

Reviewer #3: Minor revision

Answer: Additional sentences added now.

**Summary and General Comments**


Use this section to provide overall comments, discuss strengths/weaknesses of the study, novelty, significance, general execution and scholarship. You may also include additional comments for the author, including concerns about dual publication, research ethics, or publication ethics. If requesting major revision, please articulate the new experiments that are needed.

Reviewer #2: (No Response)

Reviewer #3: Overall good study and helpful for publication. However needs a more clearly stated conclusion, practical implications and recommendations.

Answer: Additional sentences added to comply with reviewer’s suggestions.
