# Supplementary material for: Evaluating the risk of dapsone hypersensitivity syndrome in Nepalese Leprosy Patients via HLA-B* 13:01 screening using real-time PCR and comparative meta-analysis of international data
Source: PLoS Negl Trop Dis. 2026 Aug 13;20(8):e0014568. doi: 10.1371/journal.pntd.0014568 (PMC13472375; doi:10.1371/journal.pntd.0014568)
Supplement: Supplementary file 5 [file pntd.0014568.s011.docx]

Dear Editors

Greetings!

Apologies for the delay in answering the comments raised during the major review.

We would like to provide our feedback as below:

Authors

17 May 2026

**Journal Requirements:**

1) Please ensure that the CRediT author contributions listed for every co-author are completed accurately and in full.
At this stage, the following Authors/Authors require contributions: Divya Raj Shamsher J. B. Rana, Mahesh Shah, Suwash Baral, Reejana Shrestha, Kishor Koju, Kanchha Shrestha, Preeti Maharjan, Jarina Joshi, Indra Bahadur Napit, Pushpendra Singh, Hana Krismawati, and Deanna A. Hagge. Please ensure that the full contributions of each author are acknowledged in the "Add/Edit/Remove Authors" section of our submission form.
The list of CRediT author contributions may be found here: <https://journals.plos.org/plosntds/s/authorship#loc-author-contributions>

Ans: Please see the author contributions section in the manuscript. Also made changes in the online submission platform.

2) Please provide an Author Summary. This should appear in your manuscript between the Abstract (if applicable) and the Introduction, and should be 150-200 words long. The aim should be to make your findings accessible to a wide audience that includes both scientists and non-scientists. Sample summaries can be found on our website under Submission Guidelines:
<https://journals.plos.org/plosntds/s/submission-guidelines#loc-parts-of-a-submission>

Ans: Now added.

3) We do not publish any copyright or trademark symbols that usually accompany proprietary names, eg ©,  ®, or TM  (e.g. next to drug or reagent names). Therefore please remove all instances of trademark/copyright symbols throughout the text, including:
- ® on page: 6.
- TM on page: 5.

Ans: Done

4) Please upload all main figures as separate Figure files in .tif or .eps format. For more information about how to convert and format your figure files please see our guidelines: 
<https://journals.plos.org/plosntds/s/figures>

Ans: Done

5) We have noticed that you have uploaded Supporting Information files, but you have not included a list of legends. Please add a full list of legends for your Supporting Information files after the references list.

Ans: We have uploaded supporting information file and supplementary files .

6) Some material included in your submission may be copyrighted. According to PLOSu2019s copyright policy, authors who use figures or other material (e.g., graphics, clipart, maps) from another author or copyright holder must demonstrate or obtain permission to publish this material under the Creative Commons Attribution 4.0 International (CC BY 4.0) License used by PLOS journals. Please closely review the details of PLOSu2019s copyright requirements here: PLOS Licenses and Copyright. If you need to request permissions from a copyright holder, you may use PLOS's Copyright Content Permission form.
Please respond directly to this email and provide any known details concerning your material's license terms and permissions required for reuse, even if you have not yet obtained copyright permissions or are unsure of your material's copyright compatibility. Once you have responded and addressed all other outstanding technical requirements, you may resubmit your manuscript within Editorial Manager. 
Potential Copyright Issues:

Ans: We have not used other’s materials.

- Please confirm (a) that you are the photographer of Figure 2., or (b) provide written permission from the photographer to publish the photo(s) under our CC BY 4.0 license.

Ans: The photo was taken by the first author.

- Figure 1: Please (a) provide a direct link to the base layer of the map (i.e., the country or region border shape) and ensure this is also included in the figure legend; and (b) provide a link to the terms of use / license information for the base layer image or shapefile. We cannot publish proprietary or copyrighted maps (e.g. Google Maps, Mapquest) and the terms of use for your map base layer must be compatible with our CC BY 4.0 license.
Note: if you created the map in a software program like R or ArcGIS, please locate and indicate the source of the basemap shapefile onto which data has been plotted.
If your map was obtained from a copyrighted source please amend the figure so that the base map used is from an openly available source. Alternatively, please provide explicit written permission from the copyright holder granting you the right to publish the material under our CC BY 4.0 license.
If you are unsure whether you can use a map or not, please do reach out and we will be able to help you. The following websites are good examples of where you can source open access or public domain maps:
* U.S. Geological Survey (USGS) - All maps are in the public domain. (http://www.usgs.gov)
* PlaniGlobe - All maps are published under a Creative Commons license so please cite u201cPlaniGlobe, http://www.planiglobe.com, CC BY 2.0u201d in the image credit after the caption. (http://www.planiglobe.com/?lang=enl)
* Natural Earth - All maps are public domain. (<http://www.naturalearthdata.com/about/terms-of-use/>).

Answer: Sorry, please find the required information below.

Base layer: <https://download.hermes.com.np/nepal-administrative-boundary-wgs/>

We had downloaded the shapefiles in 2025 and the license statement in the downloaded file said the materials could be used without permission for non-commercial purposes:

License statement:

They are downloaded from HERMES database (https://download.hermes.com.np)

They can be used for non-commercial purposes only.

It is not allowed to redistribute, or use them for commercial purposes, without prior consent.

See the website (https://download.hermes.com.np) for more information.

These data were originally extracted from the NGIID database.

Kalapani Region boundary is NOT officially.

Hermes GIS team digitize as per boundary defined by Government of Nepal.

- The following Figure contains screenshots: Figure 4. We are not permitted to publish these under our CC-BY 4.0 license, websites are usually intellectual property and are copyrighted. This includes peripheral graphics of the web browser such as icons and button. We ask that you please remove or replace it.

Ans: It has been removed.

**Reviewers' Comments:**

Reviewer's Responses to Questions

**Key Review Criteria Required for Acceptance?**


As you describe the new analyses required for acceptance, please consider the following:


**Methods**


-Are the objectives of the study clearly articulated with a clear testable hypothesis stated?

-Is the study design appropriate to address the stated objectives?

-Is the population clearly described and appropriate for the hypothesis being tested?

-Is the sample size sufficient to ensure adequate power to address the hypothesis being tested?

-Were correct statistical analysis used to support conclusions?

-Are there concerns about ethical or regulatory requirements being met?

Reviewer #1: This is a complex study of the genetic basis for the DHS in Nepal.
The study design is appropriate.

Answer: Thank you.

Reviewer #2: The objective—assessing whether HLA-B*13:01 screening can identify leprosy patients at increased risk of dapsone hypersensitivity syndrome (DHS) in Nepal and contextualising this with international evidence—is important and appropriate for PLOS NTDs. The case–control design is suitable for a pharmacogenetic association question, and the use of a rapid PCR-based assay with NGS verification is potentially impactful for programme-level risk mitigation. However, major revisions are required because core elements of the methods are currently insufficiently defined to support reproducibility and unbiased inference. The manuscript needs a clearly articulated, testable hypothesis (primary endpoint and effect measure), an explicit analytic plan, and tighter definitions of cases and controls. In particular: (i) DHS diagnostic criteria and adjudication should be described in reproducible detail (including how “new” vs historical DHS was handled and whether DRESS scoring was applied consistently); (ii) control definition needs strengthening—controls should be demonstrably comparable to cases by treatment era and exposure window, with an explicit index date and minimum follow-up to rule out delayed reactions; (iii) the “duration of MDT” variable requires a clear operational definition because the values reported are implausible for standard MDT and suggest misclassification or mixed definitions; (iv) sample size is modest, which is acceptable given large effects reported in prior literature, but the manuscript should include a brief power/precision statement and emphasise confidence intervals rather than point estimates alone; (v) the qPCR assay protocol was modified (replacement mastermix), which makes assay performance reporting essential (thresholds, QC, repeat testing rules, indeterminate handling, contamination controls, instrument model). Ethical reporting appears present (ethics approval and informed consent), but the methods should explicitly state consent procedures for retrospective DHS cases and how participant confidentiality was protected.

Answer:

(i) Done. Please see sub-sections “Dapsone Hypersensitivity (DHS) Diagnosis” and “DHS cases and Dapsone-Tolerant Controls” of Methods section for details.

(ii) Now improved. Statistical method for matching is mentioned in Methods (sub-section Statistical Calculations ), and p-values are provided in Results.

(iii) Thank you. Now corrected. The duration of MDT was according to WHO 1982 and WHO 2018, now clarified. Chi-squared test was applied to check if the proportion of controls were equivalent to the time interval of cases based on available data. Additionally, a new figure was used to show distribution of MDT start dates in cases and controls. Changes in first paragraph of Results, Table 1 and Figure 1.

(iv) Confidence interval and power now included in Methods (sub-section Statistical Calculations) and Limitations (end of 9^th^ paragraph of Discussion).

(v) qPCR modification provided with more details. Sub-section “quantitative PCR (qPCR)” in Methods section. As per the issue raised by the reviewer, we would like to clarify that the Extraction Control, Positive Control and No Template Control was used in all runs. The use of new mastermix from Qiagen (Quantitect Probe PCR MAstermix, Catalog 204343, Germany) gave Ct values in the same range as for mastermix provided by the commercial kit. The Ct value was within 20-25 for both HLA-B*13:01 and beta-actin for Nalagenetics kit and Qiagen mastermix.

(vi) With regards to your concern about the enrolment of retrospective cases and confidentiality, this has now been further clarified in the text. Retrospective participants are those who returned to Anandaban hospital or satellite clinic services during the study period. Informed consent included retroactive chart review alongside a blood sample for screening analyses.

Reviewer #3: -Are the objectives of the study clearly articulated with a clear testable hypothesis stated? yes
-Is the study design appropriate to address the stated objectives? No

Ans: Please see the study design flow chart added as Figure 1 to clarify this issue. We have included statements before the Methods section to clarify the hypothesis. Other changes are made at different places in the manuscript.

-Is the population clearly described and appropriate for the hypothesis being tested? Yes

Ans: Thank you.

-Is the sample size sufficient to ensure adequate power to address the hypothesis being tested? no, not much heterogenecity and size

Ans: A study design flow chart (Figure 1) has been added to help demonstrate how the hypothesis was tested. Power discussion (in discussion section) and confidence statements are now added to exhibit appropriate sample size ranges for analyses. DHS cases that present for treatment are relatively rare, with access for sampling most commonly associated with specialty leprosy referral centers over many years. Please see the map and ethnicity data which reflect participant diversity across Nepal (table 1).

-Were correct statistical analysis used to support conclusions? unclear whether confounding variables were considered in the analysis. many other variables that could influence DHS. not many statistical significant outcomes identified.

Ans: Statistical tests for matched case control are now includued. We did not apply logistic regression for OR. Age, sex, timing, ethnicity, BI were individually matched.

Indeed, there have not been many statically significant variables in the literature identified in association with DHS. The variables herein provide for broader analyses than found in many DHS studies, in which HLA-B*13:01 is the only variable with a consistent association evidenced across international populations for up to 85% of DHS cases. In addition, DHS fits within DRESS, which is a more comprehensive field of study involving hypersensitivity reactions to very specific types of drugs. Regarding confounding variables, Richardus and Smith criteria were used to confirm DHS in active or retroactive cases vs leprosy reactions or other DHS-related adverse reactions. A separate manuscript for the association of DHS severity with Herpesviruses reflare (which has been noted in other DRESS conditions) has been recently published by us (https://doi.org/10.1002/iid3.70385). All of this emphasizes the specific importance and value of this preventative diagnostic qPCR to reduce risks for DHS development.

-Are there concerns about ethical or regulatory requirements being met? No

Answer: Thank you.

**Results**


-Does the analysis presented match the analysis plan?

-Are the results clearly and completely presented?

-Are the figures (Tables, Images) of sufficient quality for clarity?

Reviewer #1: The results are explained clearly and in great detail.

Answer: Thank you.

Reviewer #2: At present, the results section cannot be reliably interpreted because of substantial internal inconsistencies and apparent transcription/table assembly errors in the central genotype findings. The reported case/control allele frequencies and the stated diagnostic performance metrics do not consistently align, and at least one subgroup table appears corrupted (inconsistent sample sizes and values that look pasted from another column). Before any interpretation, all primary genotype counts must be audited and presented in a single unambiguous 2×2 table (DHS vs tolerant control by allele positive/negative), and every subsequent table/figure/narrative statement must match those counts. The age-based subgroup finding (HLA-positive vs HLA-negative DHS) is contradictory between the abstract/narrative and the subgroup table and must be corrected with clear denominators and missingness reporting. Figures/tables need to be regenerated from the cleaned dataset with automated checks so totals sum correctly. The analysis presented should then match a pre-specified plan: primary association (odds ratio with 95% CI), secondary diagnostic metrics (sensitivity/specificity with 95% CI), and prespecified subgroup/sensitivity analyses that address confounding by recruitment era, ethnicity/geography, and clinical variables. For the NGS validation, concordance should be summarised clearly (n tested, concordant/discordant, and how discordant alleles are treated clinically). Finally, the meta-analysis as currently described is not reproducible; the search strategy, inclusion criteria, and modelling approach must be transparent and the analysis should be re-run using a systematic approach with clear heterogeneity reporting.

Answer: Thank you for the comments

- Matching of case and controls with statistical tests are now included (Table 1).
- A 2x2 table has been added (Table 2). In this study we only used the qPCR method and thus homozygotic or heterozygotic HLA-B*13:01 was unknown. We sent a subset of HLA-B*13:01 positive and negative samples (done by qPCR) for independent NGS. The detailed typing profile has been added in table format. The Hardy Weinberg test was not done, because a representative subset of cases and controls were not analyzed by NGS.

Reviewer #3: - Does the analysis presented match the analysis plan? Yes

Answ: Thank you

-Are the results clearly and completely presented? Yes

Ans: Thank you.

-Are the figures (Tables, Images) of sufficient quality for clarity? Yes

Ans: Thank you.

**Conclusions**


-Are the conclusions supported by the data presented?

-Are the limitations of analysis clearly described?

-Do the authors discuss how these data can be helpful to advance our understanding of the topic under study?

-Is public health relevance addressed?

Reviewer #1: I was hoping for a comment as to whether screening for this HLA variant would be cost-effective or whether it would be better to remove dapsone from the standard treatment for leprosy, but I suppose that is a separate study to be done using the results of this initial study. The authors here could recommend that such a study should be done, given the mortality from DHS.

Ans: Now included. In addition, nucleic acid amplification-based detection assays for HLA-B*13:01 reported in literature, though not necessarily validated, have been referenced in Discussion (end of 8^th^ paragraph).

Yes, the feasibility of HLA screening before MDT including the costs is part of another manuscript.

Reviewer #2: The public health rationale—preventing a severe, sometimes life-threatening adverse drug reaction by pre-treatment genetic screening—is compelling and well-aligned with PLOS NTDs. However, the current conclusions are not adequately supported because the primary results contain inconsistencies and the meta-analysis is not reported to a reproducible standard. The manuscript should substantially tone down implementation claims until the genotype results are corrected, assay performance is fully described, and the control definition bias is addressed. Limitations should be expanded to include: potential selection bias in controls (treatment era and survivorship), phenotype misclassification risk for DHS, limitations of PCR allele calling (including how allele subtypes are handled), and generalisability to other Nepalese regions/ethnic groups. Once corrected, the paper could provide useful evidence to motivate prospective screening and health-economic evaluation, but it should clearly distinguish retrospective association evidence from prospective programme feasibility.

Ans: The reviewer’s comments have been addressed. To clarify misunderstandings classified as inadequacies or error, additional background from DHS and related literature has been highlighted alongside evidence and discussion.

As raised by the reviewer here and in Methods sections:

- potential selection bias in controls (treatment era and survivorship): Selection bias have been handled with more data and statistical testing in Methods and Results first paragraph. Survivorship issue is explained in Discussion 10^th^ paragraph
- phenotype misclassification risk for DHS: Phenotype misclassification issue explained in sub-section: Dapsone Hypersensitivity (DHS) Diagnosis of Methods section.
- limitations of PCR allele calling (including how allele subtypes are handled): We tried to clarify the issue. We added two separate tables. Table 2 for qPCR results and Table 4 for allele testing by NGS. Samples from participants in this study and an independent validation control were sent for NGS to validate the results of qPCR.
- generalisability to other Nepalese regions/ethnic groups: Tried to explain in Discussion 5^th^ paragraph. As Nepal is a multi-ethnic group, the patient population was mix of different ethnicities. The ethnicities, both in cases and control, separately, were similar to national ethnicities distribution. Mentioned in last line of Results 1^st^ paragraph.

Reviewer #3: -Are the conclusions supported by the data presented? yes although not strong conclusions are able to be drawn from this study
-Are the limitations of analysis clearly described? could expand more on the limitations in the analysis.
-Do the authors discuss how these data can be helpful to advance our understanding of the topic under study? yes
-Is public health relevance addressed? Yes

Ans: Thank you. These comments have been taken into consideration alongside other changes to strengthen the manuscript.

**Editorial and Data Presentation Modifications?**


Use this section for editorial suggestions as well as relatively minor modifications of existing data that would enhance clarity. If the only modifications needed are minor and/or editorial, you may wish to recommend “Minor Revision” or “Accept”.

Reviewer #1: None.

Ans: Thank you.

Reviewer #2: Standardise terminology and allele notation throughout (consistent HLA formatting; consistent DHS vs DRESS language).

Ans: Done

Provide a concise table of participant characteristics with clear denominators and missingness for each variable.

Ans: This has been done in table 1. Clarifications are also made in Results.

Define all time-related variables (index date, time on dapsone before DHS, exposure window for controls) and ensure they are clinically plausible.

Ans: Done. Included new figure (figure 2). Also clarified in Results section.

Ensure figure/table numbering is consistent and captions are self-contained.

Ans: Done

Prefer confidence intervals for all key estimates (ORs, sensitivity/specificity, AUCs if used).

Ans: Done.

Improve language and copy-editing; there are multiple typographical/formatting artefacts that reduce readability.

Ans: Done.

Reviewer #3: Plenty of grammatical, sentence structure and spelling errors that have been overlooked. The manuscript needs adequate checking. For example:
“ HLA typing has also becxo “
“(according to WHO guidelines 2018?).”
“but the prevalence for association can potentially vary as yet untested populations”
“This is within te confidence intervals of two”
“during drug hypersensitivyt “
“ line probe assay to surveil antimicrobial drug resistance ”
“for M leprae itself”
“Nepal has probably incidence between 2-3%”
“this study.. “
“considered good “

Ans: Apologies for the mistakes. This has been addressed.

**Summary and General Comments**


Use this section to provide overall comments, discuss strengths/weaknesses of the study, novelty, significance, general execution and scholarship. You may also include additional comments for the author, including concerns about dual publication, research ethics, or publication ethics. If requesting major revision, please articulate the new experiments that are needed.

Reviewer #1: None.

Reviewer #2: This manuscript addresses a high-impact, clinically actionable question for leprosy programmes: whether HLA-B*13:01 screening can mitigate the risk of DHS in Nepal. The study’s strengths include a clear translational premise, data from a setting where evidence is valuable, and an attempt at laboratory validation of a rapid screening assay. The major weaknesses in the submitted version are (i) internal inconsistencies in the central genotype results and subgroup tables, (ii) insufficiently rigorous and clearly defined control selection, (iii) incomplete assay reporting despite a protocol modification, and (iv) a meta-analysis that is not reproducible or transparently conducted. These issues are correctable but require major revision. For acceptance, the authors should (1) fully audit and correct all genotype counts and regenerate tables/figures; (2) provide a clear primary analytic plan with appropriate effect measures and confidence intervals; (3) strengthen or re-define control inclusion criteria and add sensitivity analyses restricted to comparable treatment eras/exposure windows; (4) expand qPCR methodological reporting and present a clear concordance analysis with NGS, including handling of discordant allele calls; (5) rebuild the meta-analysis using a transparent systematic approach (search strings, selection flow, inclusion criteria, model choice, heterogeneity). With these changes and compliant data sharing, the work could make a meaningful contribution.

Answer: Thank you for the constructive comments:

i) Two tables now introduced: One for qPCR results for cases and controls (heterozygotic or homozygotic data were not available as this study used qPCR method for HLA-B*13:01 detection); second for the results of NGS typing (homo or heterozygotic data available) for all 58 samples sent for typing. But not all of the enrolled cases and control samples were sent for NGS validation. A set of independent validation cohort who ere not enrolled in this study were also sent for NGS validation.
ii) Controls definitions now improved with statistical matching.
iii) Protocol modification clarified
iv) Meta-analysis redone with PRISMA guideline. Details of PRISMA provided as supplementary materials.
1. Tables added to genotype results
2. Analytic plan added with confidence intervals
3. Matching of case control performed with statistical calculations. A figure to show MDT start dates in cases and controls added.
4. qPCR reporting detailed. Concordance analysis clarified.
5. Meta-analysis re-performed
6. Data for cases and controls added as supplementary materials

Reviewer #3: This manuscript addresses an important topic with potential relevance to public health, particularly in the context of leprosy treatment and the safety profile of dapsone. However, several limitations substantially affect the strength and interpretability of the findings.

Firstly, the sample size is relatively small and appears to be relatively homogeneous, which limits the generalizability of the results. Expanding the cohort or providing justification for its representativeness would strengthen the manuscript. The study would benefit from a more thorough comparison with existing literature on dapsone hypersensitivity syndrome. Positioning the findings in the context of prior studies would help determine whether the observed results are consistent and reproducible.

Answer: Thank you for the concerns. Sample size was indeed small. We have tried to explain the causes of low sample size in Discussions- 9^th^ paragraph. We also included statement stating that despite low sample size, the estimated effect size had sufficient power.

**Cause of low sample size**: The incidence of DHS in new cases is 2-3%. The hospital has 150-200 new cases annually and the duration of study duration was only two years. In addition, as part of bigger DHS project, all new cases in our hospital had HLA-B*13:01 pre-screening performed before MDT start. All HLA-B*13:01 positive cases did not receive dapsone which further reduced DHS incidence at our study site (feasibility study manuscript prepared). Thus the study had to rely on retrospective DHS cases and cases referred from other centers.

If we understood reviewer’s question, we do not think that the enrolled participants were genetically homogenous. As shown in Table 1, both cases and controls include significant proportions of Brahmin/Chhetri, Janajati and Madhesis, the three major ethnicities in Nepal. After the Major Review request, we performed Chi-squared tests to compare ethnicity distributions separately for cases and controls with respect to 2021 Nepal Census ethnicities. The distribution for both cases and controls were not significant, this validating the representativeness. This is mentioned in last sentences of 1^st^ Result paragraph.

We also provided the statements to mention that the OR reported in our study was within the range of confidence intervals of published meta-analyses and also in the range of our own meta-analysis (last sentence of Meta-analysis result section; First sentence of 3^rd^ paragraph in Discussion).

There are also important methodological concerns. Given that patients with leprosy are often treated with multidrug regimens, it is difficult to attribute DHS specifically to dapsone without a clearer strategy to isolate its effect. Additionally, the analysis does not appear to account for potential confounding variables, which limits the ability to infer causality from the reported associations.

Answer: Thank you for your plausible concerns. As most of the cases in our study were retrospective, it may be difficult, at this time, to delineate the culprit drug for DHS/hypersensitivity, but:

- The senior dermatologist at our hospital has been treating leprosy and DHS cases for more than 3 decades now. All DHS cases were reviewed by the senior dermatologist and two other consultant dermatologists with at least 8 year of experience in our leprosy referral center. All three dermatologist discuss all complicated cases among each other. Thus, we assume the diagnosis of DHS was correct. All the patient files were revisited by the study clinicians to confirm the diagnosis for inclusion in our study.
- All the DHS cases who had only taken MDT/dapsone for the first few weeks to 1 or 2 months before cessation of dapsone for DHS, were continued with the other remaining two MDT drugs. After subsidence of DHS symptoms, all DHS cases at the study site (irrespective of having been enrolled in this study or not) are readmitted to the hospital for a “rifampicin rechallenge” under active clinical and laboratory surveillance. As clofazimine is rarely considered a risk of hypersensitivity, rechallenge of clofazimine is not performed. After that, patients receive monthly rifampicin and daily clofazimine, and/or other alternative drug(s) in case of high bacterial load multi-bacillary patients, for rest of the required treatment durations according to the WHO guidelines.
- All cases in our study were examined for alignment with the Richardus and Smith criteria (1989) and Regi-SCAR DRESS criteria.

There are no protocol to rechallenge dapsone in suspect DHS case at the study site. Thus direct evidence of the hypersensitivity to be caused due to dapsone may be absent but indirect evidence is obvious (later use of rifampicin and clofazimine). This can provide final confirmation for attribution of DHS to dapsone.

Now, we included a section to mention the institutional practice of rifampicin re-challenge. Last paragraph of sub-section: Dapsone Hypersensitivity (DHS) Diagnosis of Methods section.

Considering the reported high mortality rate, further analysis of fatal cases would be valuable. A more detailed exploration of these outcomes could provide important clinical insights and improve the impact of the study.

Answer: Yes, this could be ideal to identify the real prevalence of HLA-B13:01 in all DHS cases. But due to limitation of our study design, majority of cases enrolled being retrospective cases visiting study site during study period, it was not possible to draw blood from dead DHS cases. No study participants who were active DHS and enrolled in our study died.

But we do have some data (not mentioned in the manuscript) on DHS cases who died. Out of the three DHS cases in last few years, not enrolled in this study, one was HLA-B13:01 positive and two were negative.

Finally, the manuscript would benefit from careful language editing. There are several instances of incomplete sentences, unclear phrasing, and inconsistencies in grammar and referencing that affect readability and overall quality.

Answer: Apologies for the mistakes. We have tried to improve the sentences and inconsistencies.

In summary, while the topic is relevant and potentially impactful, significant revisions are required to address methodological limitations, strengthen the analysis, and improve the clarity of presentation.

Answer: Thank you. Tried to improve.

PLOS authors have the option to publish the peer review history of their article ([what does this mean?](https://journals.plos.org/plosntds/s/editorial-and-peer-review-process#loc-peer-review-history)). If published, this will include your full peer review and any attached files.


If you choose “no”, your identity will remain anonymous but your review may still be made public.


**Do you want your identity to be public for this peer review?** For information about this choice, including consent withdrawal, please see our [Privacy Policy](https://www.plos.org/privacy-policy).

Reviewer #1: **Yes:** Paul Saunderson

Reviewer #2: No

Reviewer #3: No

[NOTE: If reviewer comments were submitted as an attachment file, they will be attached to this email and accessible via the submission site. Please log into your account, locate the manuscript record, and check for the action link "View Attachments". If this link does not appear, there are no attachment files.]

**Figure resubmission:**

While revising your submission, we strongly recommend that you use PLOS’s NAAS tool (https://ngplosjournals.pagemajik.ai/artanalysis) to test your figure files. NAAS can convert your figure files to the TIFF file type and meet basic requirements (such as print size, resolution), or provide you with a report on issues that do not meet our requirements and that NAAS cannot fix.

After uploading your figures to PLOS’s NAAS tool - https://ngplosjournals.pagemajik.ai/artanalysis, NAAS will process the files provided and display the results in the "Uploaded Files" section of the page as the processing is complete. If the uploaded figures meet our requirements (or NAAS is able to fix the files to meet our requirements), the figure will be marked as "fixed" above. If NAAS is unable to fix the files, a red "failed" label will appear above. When NAAS has confirmed that the figure files meet our requirements, please download the file via the download option, and include these NAAS processed figure files when submitting your revised manuscript.

Answer: Now done for all figures

**Reproducibility:**

To enhance the reproducibility of your results, we recommend that authors of applicable studies deposit laboratory protocols in protocols.io, where a protocol can be assigned its own identifier (DOI) such that it can be cited independently in the future. Additionally, PLOS ONE offers an option to publish peer-reviewed clinical study protocols. Read more information on sharing protocols at https://plos.org/protocols?utm_medium=editorial-email&utm_source=authorletters&utm_campaign=protocols

Answer: The protocol is simple thus not required at this time.

*In compliance with data protection regulations, you may request that we remove your personal registration details at any time.* [*(Remove my information/details)*](https://www.editorialmanager.com/pntd/login.asp?a=r)*. Please contact the publication office if you have any questions.*
