# Supplementary material for: Evaluating the risk of dapsone hypersensitivity syndrome in Nepalese Leprosy Patients via HLA-B* 13:01 screening using real-time PCR and comparative meta-analysis of international data
Source: PLoS Negl Trop Dis. 2026 Aug 13;20(8):e0014568. doi: 10.1371/journal.pntd.0014568 (PMC13472375; doi:10.1371/journal.pntd.0014568)
Supplement: Peer review history

# Peer review: Decision Letter 0

**DOI:** 10.1371/journal.pntd.0014568.r001

**Authors:** Stuart Blacksell, Anil Fastenau

26 Feb 2026

Evaluating the Risk of Dapsone Hypersensitivity Syndrome in Nepalese Leprosy Patients via HLA-B*13:01 Screening Using Real-Time PCR and Comparative Meta-Analysis of International Data

PLOS Neglected Tropical Diseases

Dear Dr. J. B. Rana,

Thank you for submitting your manuscript to PLOS Neglected Tropical Diseases. After careful consideration, we feel that it has merit but does not fully meet PLOS Neglected Tropical Diseases's publication criteria as it currently stands. Therefore, we invite you to submit a revised version of the manuscript that addresses the points raised during the review process.

Please submit your revised manuscript within by Apr 27 2026 11:59PM. If you will need more time than this to complete your revisions, please reply to this message or contact the journal office at plosntds@plos.org. When you're ready to submit your revision, log on to https://www.editorialmanager.com/pntd/ and select the 'Submissions Needing Revision' folder to locate your manuscript file.

Please include the following items when submitting your revised manuscript:

* A letter that responds to each point raised by the editor and reviewer(s). You should upload this letter as a separate file labeled 'Response to Reviewers'. This file does not need to include responses to any formatting updates and technical items listed in the 'Journal Requirements' section below.

* A marked-up copy of your manuscript that highlights changes made to the original version. You should upload this as a separate file labeled 'Revised Manuscript with Track Changes'.

* An unmarked version of your revised paper without tracked changes. You should upload this as a separate file labeled 'Manuscript'.

If you would like to make changes to your financial disclosure, competing interests statement, or data availability statement, please make these updates within the submission form at the time of resubmission. Guidelines for resubmitting your figure files are available below the reviewer comments at the end of this letter.

We look forward to receiving your revised manuscript.

Kind regards,

Anil Fastenau, M.D., M.Sc.

Guest Editor

PLOS Neglected Tropical Diseases

Stuart Blacksell

Section Editor

PLOS Neglected Tropical Diseases

Shaden Kamhawi

co-Editor-in-Chief

PLOS Neglected Tropical Diseases

orcid.org/0000-0003-4304-636XX

Paul Brindley

co-Editor-in-Chief

PLOS Neglected Tropical Diseases

orcid.org/0000-0003-1765-0002

**Journal Requirements:**

1) Please ensure that the CRediT author contributions listed for every co-author are completed accurately and in full.

At this stage, the following Authors/Authors require contributions: Divya Raj Shamsher J. B. Rana, Mahesh Shah, Suwash Baral, Reejana Shrestha, Kishor Koju, Kanchha Shrestha, Preeti Maharjan, Jarina Joshi, Indra Bahadur Napit, Pushpendra Singh, Hana Krismawati, and Deanna A. Hagge. Please ensure that the full contributions of each author are acknowledged in the "Add/Edit/Remove Authors" section of our submission form.

The list of CRediT author contributions may be found here: https://journals.plos.org/plosntds/s/authorship#loc-author-contributions

2) Please provide an Author Summary. This should appear in your manuscript between the Abstract (if applicable) and the Introduction, and should be 150-200 words long. The aim should be to make your findings accessible to a wide audience that includes both scientists and non-scientists. Sample summaries can be found on our website under Submission Guidelines:

https://journals.plos.org/plosntds/s/submission-guidelines#loc-parts-of-a-submission

3) We do not publish any copyright or trademark symbols that usually accompany proprietary names, eg ©,  ®, or TM  (e.g. next to drug or reagent names). Therefore please remove all instances of trademark/copyright symbols throughout the text, including:

- ® on page: 6.

- TM on page: 5.

4) Please upload all main figures as separate Figure files in .tif or .eps format. For more information about how to convert and format your figure files please see our guidelines:

https://journals.plos.org/plosntds/s/figures

5) We have noticed that you have uploaded Supporting Information files, but you have not included a list of legends. Please add a full list of legends for your Supporting Information files after the references list.

6) Some material included in your submission may be copyrighted. According to PLOSu2019s copyright policy, authors who use figures or other material (e.g., graphics, clipart, maps) from another author or copyright holder must demonstrate or obtain permission to publish this material under the Creative Commons Attribution 4.0 International (CC BY 4.0) License used by PLOS journals. Please closely review the details of PLOSu2019s copyright requirements here: PLOS Licenses and Copyright. If you need to request permissions from a copyright holder, you may use PLOS's Copyright Content Permission form.

Please respond directly to this email and provide any known details concerning your material's license terms and permissions required for reuse, even if you have not yet obtained copyright permissions or are unsure of your material's copyright compatibility. Once you have responded and addressed all other outstanding technical requirements, you may resubmit your manuscript within Editorial Manager.

Potential Copyright Issues:

- Please confirm (a) that you are the photographer of Figure 2., or (b) provide written permission from the photographer to publish the photo(s) under our CC BY 4.0 license.

- Figure 1: Please (a) provide a direct link to the base layer of the map (i.e., the country or region border shape) and ensure this is also included in the figure legend; and (b) provide a link to the terms of use / license information for the base layer image or shapefile. We cannot publish proprietary or copyrighted maps (e.g. Google Maps, Mapquest) and the terms of use for your map base layer must be compatible with our CC BY 4.0 license.

Note: if you created the map in a software program like R or ArcGIS, please locate and indicate the source of the basemap shapefile onto which data has been plotted.

If your map was obtained from a copyrighted source please amend the figure so that the base map used is from an openly available source. Alternatively, please provide explicit written permission from the copyright holder granting you the right to publish the material under our CC BY 4.0 license.

If you are unsure whether you can use a map or not, please do reach out and we will be able to help you. The following websites are good examples of where you can source open access or public domain maps:

* U.S. Geological Survey (USGS) - All maps are in the public domain. (http://www.usgs.gov)

* PlaniGlobe - All maps are published under a Creative Commons license so please cite u201cPlaniGlobe, http://www.planiglobe.com, CC BY 2.0u201d in the image credit after the caption. (http://www.planiglobe.com/?lang=enl)

* Natural Earth - All maps are public domain. (http://www.naturalearthdata.com/about/terms-of-use/).

- The following Figure contains screenshots: Figure 4.. We are not permitted to publish these under our CC-BY 4.0 license, websites are usually intellectual property and are copyrighted.This includes peripheral graphics of the web browser such as icons and button. We ask that you please remove or replace it.

**Reviewers' Comments:**

Reviewer's Responses to Questions

**Key Review Criteria Required for Acceptance?**

As you describe the new analyses required for acceptance, please consider the following:

**Methods**

-Are the objectives of the study clearly articulated with a clear testable hypothesis stated?

-Is the study design appropriate to address the stated objectives?

-Is the population clearly described and appropriate for the hypothesis being tested?

-Is the sample size sufficient to ensure adequate power to address the hypothesis being tested?

-Were correct statistical analysis used to support conclusions?

-Are there concerns about ethical or regulatory requirements being met?

Reviewer #1: This is a complex study of the genetic basis for the DHS in Nepal.

The study design is appropriate.

Reviewer #2: The objective—assessing whether HLA-B*13:01 screening can identify leprosy patients at increased risk of dapsone hypersensitivity syndrome (DHS) in Nepal and contextualising this with international evidence—is important and appropriate for PLOS NTDs. The case–control design is suitable for a pharmacogenetic association question, and the use of a rapid PCR-based assay with NGS verification is potentially impactful for programme-level risk mitigation. However, major revisions are required because core elements of the methods are currently insufficiently defined to support reproducibility and unbiased inference. The manuscript needs a clearly articulated, testable hypothesis (primary endpoint and effect measure), an explicit analytic plan, and tighter definitions of cases and controls. In particular: (i) DHS diagnostic criteria and adjudication should be described in reproducible detail (including how “new” vs historical DHS was handled and whether DRESS scoring was applied consistently); (ii) control definition needs strengthening—controls should be demonstrably comparable to cases by treatment era and exposure window, with an explicit index date and minimum follow-up to rule out delayed reactions; (iii) the “duration of MDT” variable requires a clear operational definition because the values reported are implausible for standard MDT and suggest misclassification or mixed definitions; (iv) sample size is modest, which is acceptable given large effects reported in prior literature, but the manuscript should include a brief power/precision statement and emphasise confidence intervals rather than point estimates alone; (v) the qPCR assay protocol was modified (replacement mastermix), which makes assay performance reporting essential (thresholds, QC, repeat testing rules, indeterminate handling, contamination controls, instrument model). Ethical reporting appears present (ethics approval and informed consent), but the methods should explicitly state consent procedures for retrospective DHS cases and how participant confidentiality was protected.

Reviewer #3: -Are the objectives of the study clearly articulated with a clear testable hypothesis stated? yes

-Is the study design appropriate to address the stated objectives? no

-Is the population clearly described and appropriate for the hypothesis being tested? yes

-Is the sample size sufficient to ensure adequate power to address the hypothesis being tested? no, not much heterogenecity and size

-Were correct statistical analysis used to support conclusions? unclear whether confounding variables were considered in the analysis. many other variables that could influence DHS. not many statistical significant outcomes identified.

-Are there concerns about ethical or regulatory requirements being met? no

**********

**Results**

-Does the analysis presented match the analysis plan?

-Are the results clearly and completely presented?

-Are the figures (Tables, Images) of sufficient quality for clarity?

Reviewer #1: The results are explained clearly and in great detail.

Reviewer #2: At present, the results section cannot be reliably interpreted because of substantial internal inconsistencies and apparent transcription/table assembly errors in the central genotype findings. The reported case/control allele frequencies and the stated diagnostic performance metrics do not consistently align, and at least one subgroup table appears corrupted (inconsistent sample sizes and values that look pasted from another column). Before any interpretation, all primary genotype counts must be audited and presented in a single unambiguous 2×2 table (DHS vs tolerant control by allele positive/negative), and every subsequent table/figure/narrative statement must match those counts. The age-based subgroup finding (HLA-positive vs HLA-negative DHS) is contradictory between the abstract/narrative and the subgroup table and must be corrected with clear denominators and missingness reporting. Figures/tables need to be regenerated from the cleaned dataset with automated checks so totals sum correctly. The analysis presented should then match a pre-specified plan: primary association (odds ratio with 95% CI), secondary diagnostic metrics (sensitivity/specificity with 95% CI), and prespecified subgroup/sensitivity analyses that address confounding by recruitment era, ethnicity/geography, and clinical variables. For the NGS validation, concordance should be summarised clearly (n tested, concordant/discordant, and how discordant alleles are treated clinically). Finally, the meta-analysis as currently described is not reproducible; the search strategy, inclusion criteria, and modelling approach must be transparent and the analysis should be re-run using a systematic approach with clear heterogeneity reporting.

Reviewer #3: - Does the analysis presented match the analysis plan? yes

-Are the results clearly and completely presented? yes

-Are the figures (Tables, Images) of sufficient quality for clarity? yes

**********

**Conclusions**

-Are the conclusions supported by the data presented?

-Are the limitations of analysis clearly described?

-Do the authors discuss how these data can be helpful to advance our understanding of the topic under study?

-Is public health relevance addressed?

Reviewer #1: I was hoping for a comment as to whether screening for this HLA variant would be cost-effective or whether it would be better to remove dapsone from the standard treatment for leprosy, but I suppose that is a separate study to be done using the results of this initial study. The authors here could recommend that such a study should be done, given the mortality from DHS.

Reviewer #2: The public health rationale—preventing a severe, sometimes life-threatening adverse drug reaction by pre-treatment genetic screening—is compelling and well-aligned with PLOS NTDs. However, the current conclusions are not adequately supported because the primary results contain inconsistencies and the meta-analysis is not reported to a reproducible standard. The manuscript should substantially tone down implementation claims until the genotype results are corrected, assay performance is fully described, and the control definition bias is addressed. Limitations should be expanded to include: potential selection bias in controls (treatment era and survivorship), phenotype misclassification risk for DHS, limitations of PCR allele calling (including how allele subtypes are handled), and generalisability to other Nepalese regions/ethnic groups. Once corrected, the paper could provide useful evidence to motivate prospective screening and health-economic evaluation, but it should clearly distinguish retrospective association evidence from prospective programme feasibility.

Reviewer #3: -Are the conclusions supported by the data presented? yes although not strong conclusions are able to be drawn from this study

-Are the limitations of analysis clearly described? could expand more on the limitations in the analysis.

-Do the authors discuss how these data can be helpful to advance our understanding of the topic under study? yes

-Is public health relevance addressed? yes

**********

**Editorial and Data Presentation Modifications?**

Use this section for editorial suggestions as well as relatively minor modifications of existing data that would enhance clarity. If the only modifications needed are minor and/or editorial, you may wish to recommend “Minor Revision” or “Accept”.

Reviewer #1: None.

Reviewer #2: Standardise terminology and allele notation throughout (consistent HLA formatting; consistent DHS vs DRESS language).

Provide a concise table of participant characteristics with clear denominators and missingness for each variable.

Define all time-related variables (index date, time on dapsone before DHS, exposure window for controls) and ensure they are clinically plausible.

Ensure figure/table numbering is consistent and captions are self-contained.

Prefer confidence intervals for all key estimates (ORs, sensitivity/specificity, AUCs if used).

Improve language and copy-editing; there are multiple typographical/formatting artefacts that reduce readability.

Reviewer #3: Plenty of grammatical, sentence structure and spelling errors that have been overlooked. The manuscript needs adequate checking. For example:

“ HLA typing has also becxo “

“(according to WHO guidelines 2018?).”

“but the prevalence for association can potentially vary as yet untested populations”

“This is within te confidence intervals of two”

“during drug hypersensitivyt “

“ line probe assay to surveil antimicrobial drug resistance ”

“for M leprae itself”

“Nepal has probably incidence between 2-3%”

“this study.. “

“considered good “

**********

**Summary and General Comments**

Use this section to provide overall comments, discuss strengths/weaknesses of the study, novelty, significance, general execution and scholarship. You may also include additional comments for the author, including concerns about dual publication, research ethics, or publication ethics. If requesting major revision, please articulate the new experiments that are needed.

Reviewer #1: None.

Reviewer #2: This manuscript addresses a high-impact, clinically actionable question for leprosy programmes: whether HLA-B*13:01 screening can mitigate the risk of DHS in Nepal. The study’s strengths include a clear translational premise, data from a setting where evidence is valuable, and an attempt at laboratory validation of a rapid screening assay. The major weaknesses in the submitted version are (i) internal inconsistencies in the central genotype results and subgroup tables, (ii) insufficiently rigorous and clearly defined control selection, (iii) incomplete assay reporting despite a protocol modification, and (iv) a meta-analysis that is not reproducible or transparently conducted. These issues are correctable but require major revision. For acceptance, the authors should (1) fully audit and correct all genotype counts and regenerate tables/figures; (2) provide a clear primary analytic plan with appropriate effect measures and confidence intervals; (3) strengthen or re-define control inclusion criteria and add sensitivity analyses restricted to comparable treatment eras/exposure windows; (4) expand qPCR methodological reporting and present a clear concordance analysis with NGS, including handling of discordant allele calls; (5) rebuild the meta-analysis using a transparent systematic approach (search strings, selection flow, inclusion criteria, model choice, heterogeneity). With these changes and compliant data sharing, the work could make a meaningful contribution.

Reviewer #3: This manuscript addresses an important topic with potential relevance to public health, particularly in the context of leprosy treatment and the safety profile of dapsone. However, several limitations substantially affect the strength and interpretability of the findings.

Firstly, the sample size is relatively small and appears to be relatively homogeneous, which limits the generalizability of the results. Expanding the cohort or providing justification for its representativeness would strengthen the manuscript. The study would benefit from a more thorough comparison with existing literature on dapsone hypersensitivity syndrome. Positioning the findings in the context of prior studies would help determine whether the observed results are consistent and reproducible.

There are also important methodological concerns. Given that patients with leprosy are often treated with multidrug regimens, it is difficult to attribute DHS specifically to dapsone without a clearer strategy to isolate its effect. Additionally, the analysis does not appear to account for potential confounding variables, which limits the ability to infer causality from the reported associations.

Considering the reported high mortality rate, further analysis of fatal cases would be valuable. A more detailed exploration of these outcomes could provide important clinical insights and improve the impact of the study.

Finally, the manuscript would benefit from careful language editing. There are several instances of incomplete sentences, unclear phrasing, and inconsistencies in grammar and referencing that affect readability and overall quality.

In summary, while the topic is relevant and potentially impactful, significant revisions are required to address methodological limitations, strengthen the analysis, and improve the clarity of presentation.

**********

PLOS authors have the option to publish the peer review history of their article (what does this mean?). If published, this will include your full peer review and any attached files.

If you choose “no”, your identity will remain anonymous but your review may still be made public.

**Do you want your identity to be public for this peer review?**  For information about this choice, including consent withdrawal, please see our Privacy Policy.

Reviewer #1: **Yes:** Paul Saunderson

Reviewer #2: No

Reviewer #3: No

[NOTE: If reviewer comments were submitted as an attachment file, they will be attached to this email and accessible via the submission site. Please log into your account, locate the manuscript record, and check for the action link "View Attachments". If this link does not appear, there are no attachment files.]

**Figure resubmission:**

**Reproducibility:**

To enhance the reproducibility of your results, we recommend that authors of applicable studies deposit laboratory protocols in protocols.io, where a protocol can be assigned its own identifier (DOI) such that it can be cited independently in the future. Additionally, PLOS ONE offers an option to publish peer-reviewed clinical study protocols. Read more information on sharing protocols at https://plos.org/protocols?utm_medium=editorial-email&utm_source=authorletters&utm_campaign=protocols

# Author comment: Author response to Decision Letter 1

**DOI:** 10.1371/journal.pntd.0014568.r002

28 May 2026

Attachment Submitted filename: 260517 Replied Major Review Rana et al. PLOS NTD dah.docx

# Peer review: Decision Letter 1

**DOI:** 10.1371/journal.pntd.0014568.r003

**Authors:** Stuart Blacksell, Anil Fastenau

18 Jun 2026

Response to Reviewers Revised Manuscript with Track Changes Manuscript

Shaden Kamhawi

co-Editor-in-Chief

PLOS Neglected Tropical Diseases

orcid.org/0000-0003-4304-636XX

Paul Brindley

co-Editor-in-Chief

PLOS Neglected Tropical Diseases

orcid.org/0000-0003-1765-0002

**Journal Requirements:**

1) Please amend the figure numbering in the manuscript and ensure that all figures are presented and cited in the correct numerical order throughout the text.

**Reviewers' comments:**

**Key Review Criteria Required for Acceptance?**

As you describe the new analyses required for acceptance, please consider the following:

**Methods**

-Are the objectives of the study clearly articulated with a clear testable hypothesis stated?

-Is the study design appropriate to address the stated objectives?

-Is the population clearly described and appropriate for the hypothesis being tested?

-Is the sample size sufficient to ensure adequate power to address the hypothesis being tested?

-Were correct statistical analysis used to support conclusions?

-Are there concerns about ethical or regulatory requirements being met?

Reviewer #2: The case–control design, qPCR-with-NGS validation, and analytic plan are appropriate for the pharmacogenetic question and were substantially clarified in this revision. One methodological point requires attention: the effect-estimate computation should be stated explicitly and applied consistently. The odds ratio appears as 50.1 (95% CI 15.0–166.6) in the text but 50.05 (95% CI 14.5–142.6) in Figure 4, and sensitivity is reported as both 77% and 76.5%. These differences are small but indicate more than one calculation method (e.g. Fisher exact vs logit/Woolf intervals). Please specify the single method used and propagate one value set throughout the manuscript and figures.

Reviewer #3: -Are the objectives of the study clearly articulated with a clear testable hypothesis stated? yes

-Is the study design appropriate to address the stated objectives? yes

-Is the population clearly described and appropriate for the hypothesis being tested? yes, although small population

-Is the sample size sufficient to ensure adequate power to address the hypothesis being tested? small population

-Were correct statistical analysis used to support conclusions? yes

-Are there concerns about ethical or regulatory requirements being met? no

**********

**Results**

-Does the analysis presented match the analysis plan?

-Are the results clearly and completely presented?

-Are the figures (Tables, Images) of sufficient quality for clarity?

Reviewer #2: The Results are clearly presented overall, but several quantitative inconsistencies should be corrected before the central findings can be interpreted unambiguously:

Table 6 column labelling is internally impossible as printed. The second column ("HLA(-)") holds group totals rather than allele-negative counts — the present study row lists 34 and 82 under "HLA(-)" when the true negatives are 8 and 77. The pooled estimates are arithmetically correct (sensitivity 193/226 = 85.4%; specificity 1161/1312 = 88.5%, with 1161 = 1312−151), so the underlying calculation holds, but the table should be relabelled "Total (n)" or repopulated with true HLA-negative counts.

The Zhang 2013 denominators disagree between Figure 5 (controls 148/2064) and Table 6 (controls 119/833). Because this is the largest contributing study, the discrepancy materially affects the pooled estimate. Please reconcile the extraction across the forest plot, table, and text.

"Chen et al. 2018" (Table 6) and "WT et al. 2018" (forest plot) appear to be the same Taiwan paper (JID 138(7):1546–54). Please confirm it is not double-counted in the pooled sensitivity and merge the reference.

Confidence intervals are inconsistent across the manuscript: PPV 1.9–43.4 (Results) vs 11.9–43.4 (Discussion); NPV 98.8–99.6 (Results) vs 8.8–99.7 (Discussion, an apparent typo). These should be harmonised.

Table 5 subgroup denominators are uneven and imply unreported missingness — fever 15/16, rashes 14/14, jaundice 17/17 against group n = 26/8 — and "Probable 93.4%" does not match a /16 or /26 denominator. Please report explicit denominators and missingness per row.

Reviewer #3: -Does the analysis presented match the analysis plan? yes

-Are the results clearly and completely presented? yes

-Are the figures (Tables, Images) of sufficient quality for clarity? yes

**********

**Conclusions**

-Are the conclusions supported by the data presented?

-Are the limitations of analysis clearly described?

-Do the authors discuss how these data can be helpful to advance our understanding of the topic under study?

-Is public health relevance addressed?

Reviewer #2: The conclusions are supported and the public-health relevance (pre-treatment screening to prevent a high-morbidity, ~10%-mortality reaction) is well-aligned with the journal. One interpretive correction: the manuscript states a negative likelihood ratio <0.1 provides strong rule-out evidence, then reports NLR = 0.25, which does not meet that threshold. As written it implies the test qualifies. The rule-out argument should rest on the prevalence-dependent NPV (99.4% at 2.5% incidence), not the NLR; please revise the wording so 0.25 is described as moderate.

Reviewer #3: -Are the conclusions supported by the data presented? needs a more explicit conclusion with recommendations and implications

-Are the limitations of analysis clearly described? yes

-Do the authors discuss how these data can be helpful to advance our understanding of the topic under study? no

-Is public health relevance addressed? yes

**********

**Editorial and Data Presentation Modifications?**

Use this section for editorial suggestions as well as relatively minor modifications of existing data that would enhance clarity. If the only modifications needed are minor and/or editorial, you may wish to recommend “Minor Revision” or “Accept”.

Reviewer #2: Minor, all tied to the consistency issues above: regenerate Tables 5 and 6 and Figures 4–5 from the cleaned dataset with row/column totals that sum correctly and self-contained captions; standardise the single OR/sensitivity value set throughout; and merge the duplicate Chen/WT 2018 reference. These are correctable as part of a minor revision.

Reviewer #3: Minor revision

**********

**Summary and General Comments**

Use this section to provide overall comments, discuss strengths/weaknesses of the study, novelty, significance, general execution and scholarship. You may also include additional comments for the author, including concerns about dual publication, research ethics, or publication ethics. If requesting major revision, please articulate the new experiments that are needed.

Reviewer #2: (No Response)

Reviewer #3: Overall good study and helpful for publication. However needs a more clearly stated conclusion, practical implications and recommendations.

**********

PLOS authors have the option to publish the peer review history of their article (what does this mean?). If published, this will include your full peer review and any attached files.

If you choose “no”, your identity will remain anonymous but your review may still be made public.

**Do you want your identity to be public for this peer review?**  For information about this choice, including consent withdrawal, please see our Privacy Policy.

Reviewer #2: No

Reviewer #3: No

**Figure resubmission:**

**Reproducibility:**

To enhance the reproducibility of your results, we recommend that authors of applicable studies deposit laboratory protocols in protocols.io, where a protocol can be assigned its own identifier (DOI) such that it can be cited independently in the future. Additionally, PLOS ONE offers an option to publish peer-reviewed clinical study protocols. Read more information on sharing protocols at https://plos.org/protocols?utm_medium=editorial-email&utm_source=authorletters&utm_campaign=protocols

# Author comment: Author response to Decision Letter 2

**DOI:** 10.1371/journal.pntd.0014568.r004

10 Jul 2026

Attachment Submitted filename: Response to Reviewers.docx

# Editor report: Decision Letter 2

**DOI:** 10.1371/journal.pntd.0014568.r005

**Authors:** Stuart Blacksell, Anil Fastenau

12 Jul 2026

Dear Mr. J. B. Rana,

We are pleased to inform you that your manuscript 'Evaluating the Risk of Dapsone Hypersensitivity Syndrome in Nepalese Leprosy Patients via HLA-B*13:01 Screening Using Real-Time PCR and Comparative Meta-Analysis of International Data' has been provisionally accepted for publication in PLOS Neglected Tropical Diseases.

Before your manuscript can be formally accepted you will need to complete some formatting changes, which you will receive in a follow up email. A member of our team will be in touch with a set of requests.

Please note that your manuscript will not be scheduled for publication until you have made the required changes, so a swift response is appreciated.

IMPORTANT: The editorial review process is now complete. PLOS will only permit corrections to spelling, formatting or significant scientific errors from this point onwards. Requests for major changes, or any which affect the scientific understanding of your work, will cause delays to the publication date of your manuscript.

Should you, your institution's press office or the journal office choose to press release your paper, you will automatically be opted out of early publication. We ask that you notify us now if you or your institution is planning to press release the article. All press must be co-ordinated with PLOS.

Thank you again for supporting Open Access publishing; we are looking forward to publishing your work in PLOS Neglected Tropical Diseases.

Best regards,

Anil Fastenau, M.D., M.Sc.

Guest Editor

PLOS Neglected Tropical Diseases

Stuart Blacksell

Section Editor

PLOS Neglected Tropical Diseases

Shaden Kamhawi

co-Editor-in-Chief

PLOS Neglected Tropical Diseases

orcid.org/0000-0003-4304-636XX

Paul Brindley

co-Editor-in-Chief

PLOS Neglected Tropical Diseases

orcid.org/0000-0003-1765-0002

***********************************************************

p.p1 {margin: 0.0px 0.0px 0.0px 0.0px; line-height: 16.0px; font: 14.0px Arial; color: #323333; -webkit-text-stroke: #323333}span.s1 {font-kerning: none

# Editor report: Acceptance letter

**DOI:** 10.1371/journal.pntd.0014568.r006

**Authors:** Stuart Blacksell, Anil Fastenau

Dear Mr. J. B. Rana,

We are delighted to inform you that your manuscript, "Evaluating the Risk of Dapsone Hypersensitivity Syndrome in Nepalese Leprosy Patients via HLA-B*13:01 Screening Using Real-Time PCR and Comparative Meta-Analysis of International Data," has been formally accepted for publication in PLOS Neglected Tropical Diseases.

We have now passed your article onto the PLOS Production Department who will complete the rest of the publication process. All authors will receive a confirmation email upon publication.

The corresponding author will soon be receiving a typeset proof for review, to ensure errors have not been introduced during production. Please review the PDF proof of your manuscript carefully, as this is the last chance to correct any scientific or type-setting errors. Please note that major changes, or those which affect the scientific understanding of the work, will likely cause delays to the publication date of your manuscript. Note: Proofs for Front Matter articles (Editorial, Viewpoint, Symposium, Review, etc...) are generated on a different schedule and may not be made available as quickly.

Soon after your final files are uploaded, the early version of your manuscript will be published online unless you opted out of this process. The date of the early version will be your article's publication date. The final article will be published to the same URL, and all versions of the paper will be accessible to readers.

For Research Articles, you will receive an invoice from PLOS for your publication fee after your manuscript has reached the completed accept phase. If you receive an email requesting payment before acceptance or for any other service, this may be a phishing scheme. Learn how to identify phishing emails and protect your accounts at https://explore.plos.org/phishing.

Thank you again for supporting open-access publishing; we are looking forward to publishing your work in PLOS Neglected Tropical Diseases.

Best regards,

Shaden Kamhawi

co-Editor-in-Chief

PLOS Neglected Tropical Diseases

Paul Brindley

co-Editor-in-Chief

PLOS Neglected Tropical Diseases
